# Enhanced Hemolytic Activity of Mesophilic *Aeromonas salmonicida* SRW-OG1 Is Brought about by Elevated Temperatures

**DOI:** 10.3390/microorganisms10102033

**Published:** 2022-10-14

**Authors:** Yunong Chen, Jiajia Wang, Hongyan Cai, Mao Lin, Youyu Zhang, Lixing Huang

**Affiliations:** 1 Key Laboratory of Healthy Mariculture for the East China Sea, Fisheries College, Jimei University, Ministry of Agriculture, Xiamen 361000, China; 2 Institute of Electromagnetics and Acoustics, School of Electronic Science and Engineering, Xiamen University, Xiamen 361000, China

**Keywords:** *Aeromonas salmonicida*, transcriptome analysis, temperature adaption, virulence regulation, *aerA*, *hlyA*

## Abstract

*Aeromonas salmonicida* is a well-known cold-water pathogenic bacterium. Previously, we reported the first isolation of pathogenic *A. salmonicida* from diseased *Epinephelus coioides*, a kind of warm-water fish, and it was proved to be a putative mesophilic strain with potent pathogenicity to humans. In order to investigate the mechanisms underlying mesophilic growth ability and virulence, the transcriptome of *A. salmonicida* SRW-OG1 at 18, 28, and 37 °C was analyzed. The transcriptome of *A. salmonicida* SRW-OG1 at different temperatures showed a clear separation boundary, which might provide valuable information for the temperature adaptation and virulence regulation of *A. salmonicida* SRW-OG1. Interestingly, *aerA* and *hlyA*, the hemolytic genes encoding aerolysin and hemolysin, were found to be significantly up-regulated at 28 and 37 °C. Since aerolysin and hemolysin are the most well-known and -characterized virulence factors of pathogenic *Aeromonas* strains, the induction of *aerA* and *hlyA* was associated with the mesophilic virulence. Further study proved that the extracellular products (ECPs) purchased from *A. salmonicida* SRW-OG1 cultured at 28 and 37 °C showed elevated hemolytic activity and virulence than those at 18 °C. Moreover, the silence of *aerA* and *hlyA* led to significantly decreased hemolysis and virulence. Taken together, our results revealed that the mesophilic virulence of *A. salmonicida* SRW-OG1 might be due to the enhanced expression of *aerA* and *hlyA* induced by elevated temperatures.

## 1. Introduction

*Aeromonas salmonicida* is pathogenetic to freshwater and marine fish. So far, there have been reports that at least rainbow trout (*Oncorhynchus mykiss*), goldfish (*Carassius auratus*), carp (*Cyprinus carpio*), Atlantic salmon (*Salmo salar*), as well as turbot (*Scophthalmus maximus* L.), have been infected with *A. salmonicida* [1,2,3,4,5,6]. *A. salmonicida*-induced furunculosis has been one of the chief threats to fish culture worldwide [7]. The symptoms of the diseased fish are bleeding fins, melanoma, blackening of skin, drowsiness, and loss of appetite [8].

It is usually considered that *A*. *salmonicida* is a well-known cold-water pathogenic bacterium [9,10]. Although it is generally believed that *A*. *salmonicida* can only grow at temperatures below 25 °C, a few strains have been found to grow effectively at 37 °C, so they are considered to be mesophilic [11,12,13,14,15]. Recent studies have shown that some *A*. *salmonicida* strains are proved to be mesophilic and capable of infecting humans and mammals [16,17]. The SRW-OG1 strain was previously isolated from orange-spotted grouper (*Epinephelus coioides*) with furunculosis. It is a Gram-negative bacterium with short rod-shaped. The result of an indole test on SRW-OG1 was positive, indicating that tryptophan can be metabolized by the strain. Previous study also showed that SRW-OG1 was mesophilic with potential pathogenicity to humans [18]. Thus, it is meaningful to explore the mechanisms of its temperature adaption and virulence regulation.

To facilitate the understanding of the mechanisms underlying the mesophilic growth ability and virulence regulation, the transcriptome of SRW-OG1 was sequenced at 18 °C (virulent temperature in cold-water fish), 28 °C (virulent temperature in warm-water fish), and 37 °C (virulent temperature in humans) in this study. Interestingly, *aerA* and *hlyA*, the hemolytic genes encoding aerolysin and hemolysin, were found to be significantly up-regulated at 28 and 37 °C.

As we know, virulence factors of pathogenic *Aeromonas* strains include proteases, aerolysin, hemolysin, enterotoxins, and acetylcholinesterase [19]. Aerolysin, a pore forming toxin with hemolytic, cytotoxic, and enterotoxic activities, plays a critical role in the pathogenicity of *Aeromonas* strains [20]. After binding to the glycosylphosphatidylinositol anchor of the host cell, the aerolysin will be concentrated and promote the formation of oligomers that form channel pores which can be inserted into the membrane [21]. Hemolysin is also a pore-forming toxin secreted by pathogenic *Aeromonas* strains [22] which is strongly associated with the virulence. The direct detection of *aerA* and *hlyA* is identified as a reliable approach for identifying potentially pathogenic *Aeromonas* strains [23].

Since aerolysin and hemolysin are the most well-known and -characterized virulence factors of pathogenic *Aeromonas* strains, the relationship between them and mesophilic virulence was worth exploring. Therefore, further evaluation of the effects of temperature and hemolytic genes (*aerA* and *hlyA*) was carried out in the present study. Our results proved that the extracellular products (ECPs) purchased from *A*. *salmonicida* SRW-OG1 cultured at 28 and 37 °C showed elevated hemolytic activity and virulence than those at 18 °C. Moreover, the silence of *aerA* and *hlyA* led to significantly decreased hemolysis and virulence. Taken together, our results revealed that the mesophilic virulence of *A*. *salmonicida* SRW-OG1 might be due to the enhanced expression of *aerA* and *hlyA* induced by elevated temperatures.

## 2. Materials and Methods

### 2.1. Sampling

The SRW-OG1 strain was isolated in Zhangzhou city from diseased *E*. *coioides* in 2020. It was cultured in Luria–Bertani (LB) medium (Sangon Biotech, Shanghai, China) at 18, 28 or 37 °C (220 rpm). For RNA extraction, bacterial cultures were adjusted to OD_560_ = 0.3 [24,25].

### 2.2. Transcriptome Sequencing

*A*. *salmonicida* SRW-OG1 was precultured overnight in LB at 18 °C (220 rpm). Then, the bacterial solution was inoculated into 5 mL of LB medium (1:100) and cultured in 10 mL centrifuge tubes for 8 h at 18, 28, or 37 °C (220 rpm), separately. For RNA extraction, OD_560_ of the bacterial culture was adjusted to 0.3. Total RNA was extracted from SRW-OG1 according to our previous description [26]. Three biological replicates were set up for each temperature. Total RNA was obtained according to the manufacturer’s instructions with Trizol^®^ reagent (Invitrogen, Waltham, MA, USA) [27]. The RNA quality was validated using 2100 Bioanalyser (Agilent, Santa Clara, CA, USA) together with ND-2000 (NanoDrop technology, Waltham, MA, USA). The following sequencing libraries were generated using high-quality RNA samples.

RNA-seq was carried out by Majorbio Biotech (Shanghai, China) using Illumina HiSeq4000 sequencer (Illumina, San Diego, CA, USA). PE150 sequencing strategy and the 250–300 bp insert-specific library were used in this study [28]. The preparation of transcriptome library was executed using TruSeqTM RNA sample kit (Illumina, San Diego, CA, USA) with 2 μg of total RNA. We performed the ribosomal RNA (rRNA) depletion after poly(A) purification by the Ribo-Zero Magnetic kit (Illumina, San Diego, CA, USA), which was followed by dissociation of each mRNA into short fragments (200 nt). After that, we synthesized the double-stranded cDNA by the SuperScript double-stranded cDNA synthesis kit (Invitrogen, Waltham, CA, USA). The dTTP was replaced by dUTP integrated with running of the second-strand cDNA. The end-repair and phosphorylation were then carried out on the newly formed cDNA. At the same time, UNG enzymes degraded the strand cDNA with dUTP. With 2% low range ultra agarose, the cDNA fragments of 200 bp were selected to contribute libraries with PCR amplification by Phusion DNA polymerase (NEB, Ipswich, MA, USA). Amplified fragments were quantified with TBS380, followed by paired-end RNA-seq libraries using Illumina HiSeq × TEN. Read data were deposited in the GSA database (PRJCA005431).

### 2.3. Read Processing and Mapping

The reference genome sequence of *A*. *salmonicida* SRW-OG1 (accession number: PRJNA622411) was used for the mapping of clean reads using SOAP2 [29]. Less than 5 mismatches were acceptable during the alignment process. The RPKM method was used to calculate the unigene expression [30]. Detection and visualization of significantly changed genes (*p*-value < 0.001, fold change ≥ 2) was carried out with the two classes unpaired MA-plot-based method. PCA analysis was performed using the PCA() from Scratch in Python.

### 2.4. Functional Classification and Enrichment Analysis

For gene annotation, we used the Blast2GO program (Blast2GO 5.2.5, BioBam Bioinformatics, Valencia, Spain) to obtain GO annotation of the unigenes. After acquiring the GO annotation for every gene, we used WEGO software(https://biodb.swu.edu.cn/cgi-bin/wego/index.pl, accessed on 15 June 2022) to carry out GO functional classification for all genes and understand the distribution of gene functions of the species at the macro level. The calculated *p*-value went through Bonferroni correction, taking corrected *p*-value ≤ 0.05 as a threshold. GO terms fulfilling this condition were defined as significantly enriched. The NR (ftp://ftp.ncbi.nlm.nih.gov/blast/db/, accessed on 23 June 2022), COG (http://www.ncbi.nlm.nih.gov/COG, accessed on 23 June 2022), Swiss-Prot (ftp://ftp.uniprot.org/pub/databases/uniprot/current_release/knowledgebase/complete/uniprot_sprot.fasta.gz, accessed on 23 June 2022), and KEGG (http://www.genome.jp/kegg/, accessed on 23 June 2022) databases were used for the gene annotations. Q-value was defined to be the FDR analogue of the *p-*value. Pathways with q-values ≤ 0.05 were identified as significant enrichment in genes.

### 2.5. Quantitative Real-Time PCR (qPCR)

The Power Green qPCR Mix (Dongsheng Biotech Co., Guangzhou, China) was used for qPCR. The total volume of the reaction is 10 μL, which includes 0.25 μL forward primer (10 μM), 0.25 μL reverse primer (10 μM), 0.5 μL template, and 9.0 μL 2× Power Green qPCR Mix (Dongsheng Biotech Co., Guangzhou, China). Subsequently, the experiment was carried out on the QuantStudio^TM^ 6 qPCR system (ABI, Waltham, MA, USA). The thermal cycler conditions were 95 °C for 2 min, followed by 40 cycles of 95 °C for 20 s, 58 °C for 20 s, and 72 °C for 20 s. All qRT-PCR experiments were performed in triplicate using independent samples. The gene expression of target genes were normalized to 16S RNA and *gyrB* with the 2^−ΔΔCt^ calculation method [31]. Primers were designed using Primer 5 software (Primer 5, Premier Biosoft International, Palo Alto, CA, USA) and listed in Table S1.

### 2.6. Sequence Alignment Analysis, Evolutionary Tree Construction, and Protein Structure Prediction

The nucleic acid sequences of the *aerA* and *hlyA* of *A*. *salmonicida* and another 4 different strains were found in NCBI (www.ncbi.nlm.nih.gov, accessed on 28 June 2022), and a phylogenetic tree was constructed using MEGA7. The corresponding amino acid sequences were obtained according to the *aerA* and *hlyA* nucleic acid sequences of 5 strains, then MEGA7 and GeneDoc were used for sequence alignment. Based on the amino acid sequence of *aerA* and *hlyA* of *A*. *salmonicida*, the secondary structures were predicted by I-TASSER (https://zhanggroup.org/I-TASSER/, accessed on 28 June 2022).

### 2.7. Knockdown of aerA and hlyA in A. salmonicida

Five knocked-down strains of *A*. *salmonicida aerA* and *hlyA* were constructed using RNAi, respectively. The method used here referred to Choi [32] and Darsigny [33], with slight modifications. The reference genome sequence of *A*. *salmonicida aerA* and *hlyA* was introduced into Invitrogen Block-iT^TM^ RNAi Designer (https://rnaidesigner.thermofisher.com/rnaiexpress/design.do, accessed on 3 July 2022) to design shRNA online. T4 DNA ligase was used to connect shRNA to linear pCM130, and the connected product was first transferred into *E*. *coli* DH5α by heat shock according to the technical method of Huang [24], and then introduced into the competence of *A*. *salmonicida* by electroporation and cultured in agar plate (LB with 100 μg/mL tetracycline) at 28 °C overnight. Positive colonies were verified by gene sequencing and the expression level of *aerA* and *hlyA* was detected by qRT-PCR. The strains displaying the best silencing efficiency were used for further study.

### 2.8. Determination of Hemolytic Ability

Hemolytic capacity of *A*. *salmonicida* was detected according to the methods described by Wei [25]. We centrifuged 100 μL of fresh sheep blood (Ping Rui Biotechnology Co., Ltd., Beijing, China) at 3000 rpm for 10 min at 25 °C, and the supernatant was discarded. Then, the erythrocytes were rinsed 3 times with 200 μL of phosphate-buffered saline and resuspended with 100 μL of phosphate-buffered saline. We mixed 5 μL of the resuspended erythrocytes with 100 µL of trypsin-treated bacterial supernatants and incubated for 1 h at 37 °C, 150 rpm. Lastly, the mixture was centrifuged at 5000 rpm for 3 min and 100 μL of the supernatant was used to determine OD_540_.

### 2.9. Extraction of ECPs at Different Culture Temperatures

ECPs were prepared as per the previous description [34] with minor modification. To be specific, bacteria were inoculated in LB medium and placed in an air bath shaker at 18 °C and 120 r/min for 24 h. We evenly spread 200 μL bacterial suspension on LB plate pre-covered with a layer of sterile cellophane, and cultured at 18, 28, or 37 °C for 48 h. Each plate was washed with 4 mL sterile PBS, and the washed bacterial suspension was collected and centrifuged at 4 °C for 30 min at 12,000 r/min. The supernatant was taken and frozen to solid state at −80 °C. After that, the supernatant was put into vacuum freeze dryer and lyophilized. An appropriate amount of the powdered sample was dissolved with sterile PBS, and then filtered by 0.22 µm membrane for sterilization to obtain the crude extracellular product. Total protein content of supernatants was determined by the Bradford method. Before use, it was confirmed aseptic growth and the initial protein concentration was adjusted to 10 μg/μL.

### 2.10. Artificial Infection and Sampling

All animal experiments were carried out to refer to the ‘Guide for the Care and Use of Laboratory Animals’ set by the Animal Ethics Committee of Jimei University (Acceptance NO JMULAC201159).

Artificial infection was performed according to the method of Huang with slight modifications. Healthy and disease-free *E*. *coioides* (42.0 ± 1.0 g) obtained from Zhangzhou, Fujian Province (China), were randomly divided into groups (15 /group, set up with three replicates) and kept in a pre-disinfected laboratory for one week. Fish were tested to be healthy by sera agglutination and bacteriological recovery tests, as described by Huang et al. [35]. *E*. *coioides* was intraperitoneally injected with 200 μL 10^3^ CFU/g *A*. *salmonicida* SRW-OG1 suspension, and an equal volume of phosphate-buffered saline (PBS) was intraperitoneally injected as a negative control. The morbidity and mortality of *E*. *coioides* were observed daily [35].

The toxicity of ECPs to *E*. *coioides* from *A*. *salmonicida* cultured at 18, 28, and 37 °C was validated according to the previous description [35]. *E*. *coioides* was intraperitoneally injected with 100 μL of each preparation mentioned above, and an equal volume of PBS was intraperitoneally injected as a negative control. The morbidity and mortality of *E*. *coioides* were observed daily.

### 2.11. Statistical Analysis

Data were presented as mean ± standard deviation (SD) and analyzed by SPSS 24.0 software (IBM, Armonk, NY, USA). Differences were compared by *t*-test, one-way ANOVA followed by Dunnett’s test. *p* < 0.05 was considered statistically significant [36].

## 3. Results

### 3.1. RNA and Data Quality

**Figure 1:**
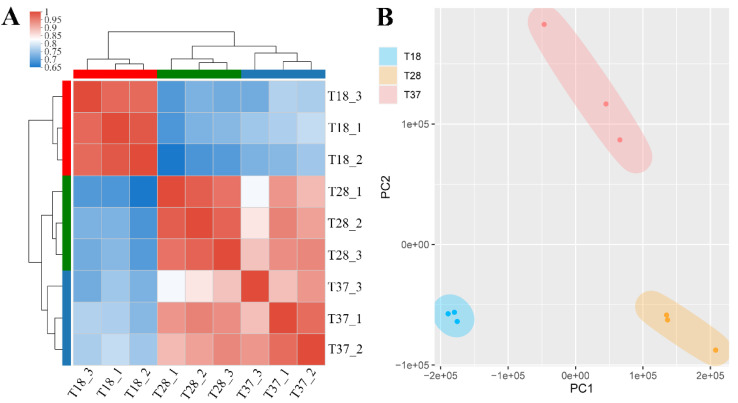
Evaluation of sample variance. (**A**) Heatmap showing the sample-to-sample distance (the squares with different colors represent the correlation between two samples). (**B**) Principal component analysis of transcripts from *A*. *salmonicida* inoculated at 18 °C (blue), 28 °C (orange) and 37 °C (red).

On average, RNA-seq generated 25,250,066 high-quality reads per sample, approximately 96.8% of which mapped uniquely to the genome of SRW-OG1 (accession number: PRJNA622411) (Table S2). The samples were clustered mainly by individuals and treatments, as indicated by the correlation matrix of gene expression (Figure 1A). PCA analysis revealed that when incubated at different temperatures, samples showed clear separation, whereas samples from the same group generally clustered together (Figure 1B).

### 3.2. Functional Annotation of Genes Identified in All Samples

To have an overall understanding of the expressible genes carried by this strain, functional annotation of genes identified in all samples was carried out. Totally, 4088 genes were functionally annotated by public databases, including NR, Swiss-Prot, COG, and KEGG. Among them, 1843 genes were functionally annotated by these databases simultaneously (Figure S1). According to the KEGG database, genes were assigned to 41 KEGG pathways (Figure S2). According to the GO database, genes were categorized into 27 enriched functional groups (Figure S3).

### 3.3. Validation of RNA-seq

**Figure 2:**
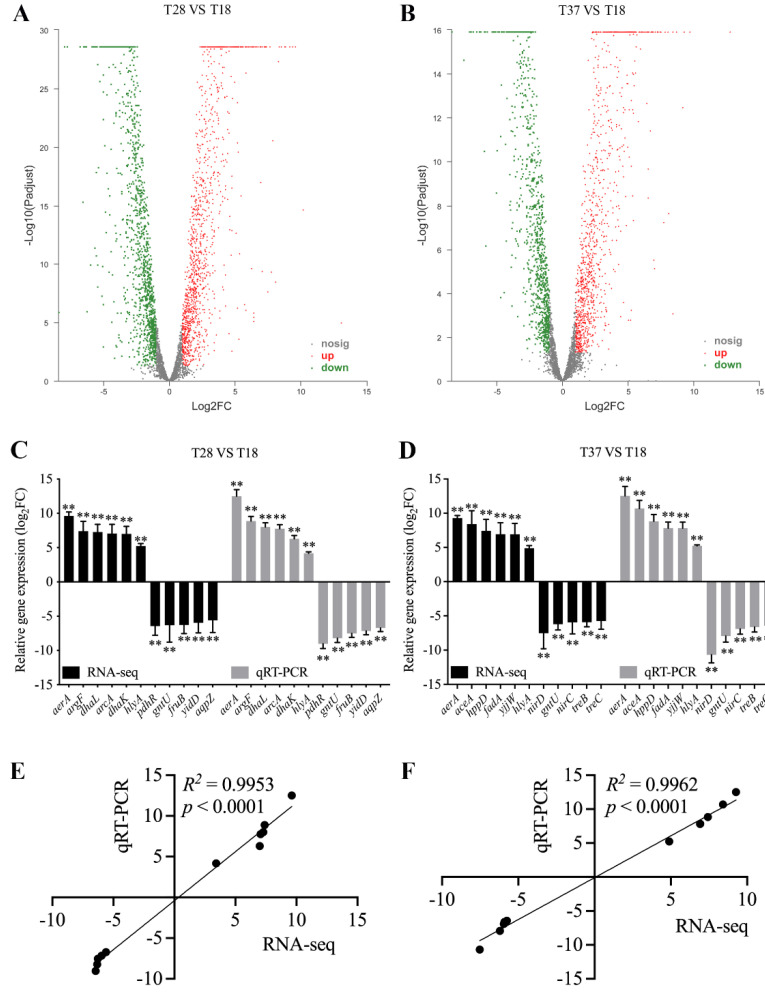
Validation of gene expression. (**A**,**B**) Volcano plot of all genes at 28 °C (**A**) and 37 °C (**B**) compared to those at 18 °C; *X*-axis represents the fold change values, *Y*-axis represents statistical test value. Red dots indicate genes significantly up-regulated. Green dots represent genes significantly down-regulated. Grey dots represent genes non-significantly regulated. (**C**,**D**) qPCR validation of genes randomly selected at 28 °C (**C**) and 37 °C (**D**) compared to those at 18 °C. Data are presented as mean ± SD (*n* = 3). ** *p* < 0.01 means a significant difference from the 18 °C group. (**E**) Correlation analysis of expression levels between 18 and 28 °C identified by qPCR and RNA-seq. (**F**) Correlation analysis of expression levels between 18 and 37 °C identified by qPCR and RNA-seq. A value of *p* < 0.05 is used to indicate significant correlations.

Compared to the 18 °C-treated group, a significant regulation of 2499 genes (1242 up-regulated and 1257 down-regulated) was observed in *A*. *salmonicida* SRW-OG1 grown at 28 °C (Figure 2A). Compared to the 18 °C-treated group, a significant regulation of 2021 genes (1060 up-regulated and 961 down-regulated) was observed in *A*. *salmonicida* SRW-OG1 grown at 37 °C (Figure 2B). The differentially expressed genes are listed in Table S3. For further validation of RNA-seq, qPCR was carried out on randomly selected differential expressed genes. The qPCR results displayed similar trends as the RNA-seq results (Figure 2C,D). The correlations tests showed that the expression levels detected by qPCR were significantly associated with those detected by RNA-seq (Figure 2E,F). These further proved the reliability of RNA-seq results.

### 3.4. Analysis of Differentially Expressed Genes

**Figure 3:**
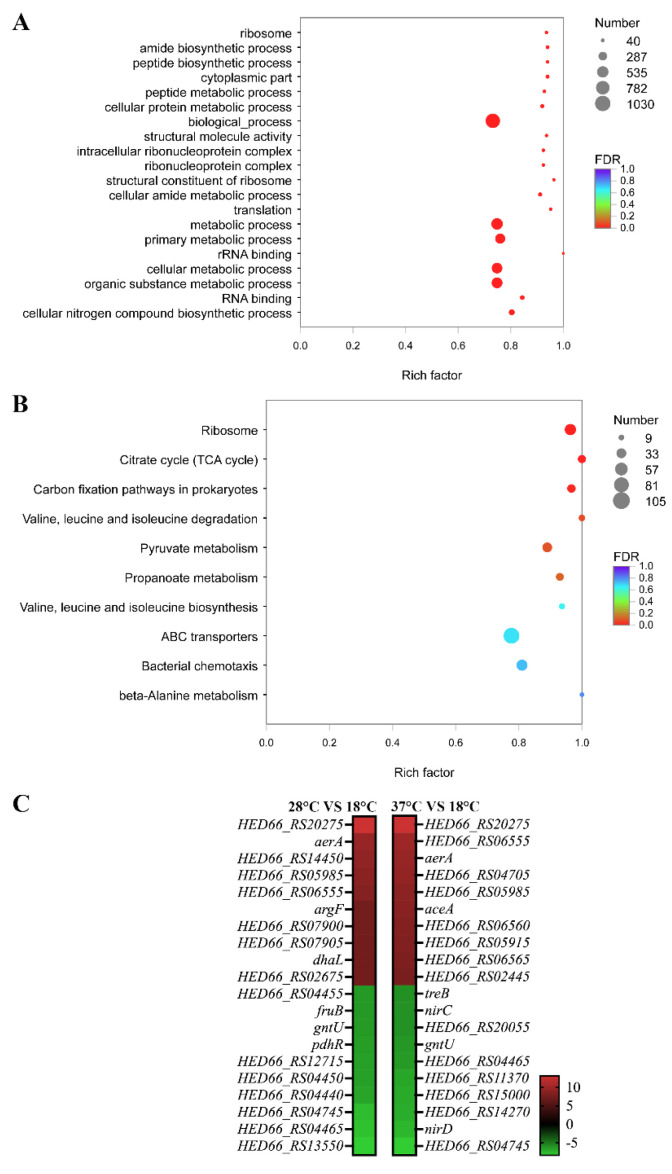
Analysis of differentially expressed genes. (**A**) GO terms of all the DEGs were classified based on their molecular functions, involvement of biological processes, and cellular components. The size of the dot indicates the number of genes/transcripts in this GO term, and the color of the dot corresponds to different FDR ranges. (**B**) KEGG enrichment of all DEGs. The size of the dot indicates the number of genes in this pathway, and the color of the dot corresponds to different q-value ranges. (**C**) Heat map of the 20 genes most significantly affected at 28 °C or 37 °C (10 genes with the highest up-regulated ratio and 10 genes with the highest down-regulated ratio). Values represent log2 folds. Colors based on log-transformed transcripts FPKM mean values. Blue and red indicate decreased and increased expression, respectively.

The 2784 differentially expressed genes were clustered hierarchically, thus generating the heatmap (Figure S4A). The heatmap not only showed their changing trends, but also revealed the distinguishable expression profiles among samples incubated at different temperatures. GO enrichment on these DEGs was then performed, in which several processes showed significant enrichment of DEGs, such as “metabolic process”, “RNA binding”, and “intracellular ribonucleoprotein complex” (Figure 3A). We also mapped the 2784 DEGs to the reference canonical pathway in KEGG (Figure 3B). “Ribosome”, “TCA cycle”, “Carbon fixation pathways in prokaryotes”, and “Valine, leucine, and isoleucine degradation” were the most significant enriched pathways of DEG. Equally representative pathways were “Pyruvate metabolism”, “Propanoate metabolism”, and “Valine, leucine, and isoleucine biosynthesis”. As we can see, most of the affected pathways were related to cellular metabolism. Among the enriched KEGG pathways, there were some known pathways closely related to environmental adaptation and virulence regulation, for example: “ABC transporters” and “Bacterial chemotaxis”. There were 105 genes enriched in “ABC transporters”, such as *znuABC*, *oppABCDF*, and *afuABC*. There were 51 genes enriched in “Bacterial chemotaxis”, such as *mcp*, *fliG*, *motA*, *cheB*, *cheY*, and *cheV*.

We selected the 20 genes most significantly affected at 28 °C or 37 °C (10 genes with the highest up-regulated ratio and 10 genes with the highest down-regulated ratio) (Figure 3C). The function of some of these genes, such as HED66_RS20275, HED66_RS14450, HED66_RS05985, and HED66_RS06555, are still unclear. Other genes have been reported to be more or less related to bacterial pathogenicity, such as *aerA*, *argF*, *dhaL*, *fruB*, *gntU*, *pdhR*, *aceA*, *treB*, *nirC,* and *nirD*. Interestingly, *aerA* and *hlyA*, the hemolytic genes encoding aerolysin and hemolysin, were found to be significantly up-regulated at 28 and 37 °C.

**Figure 4:**
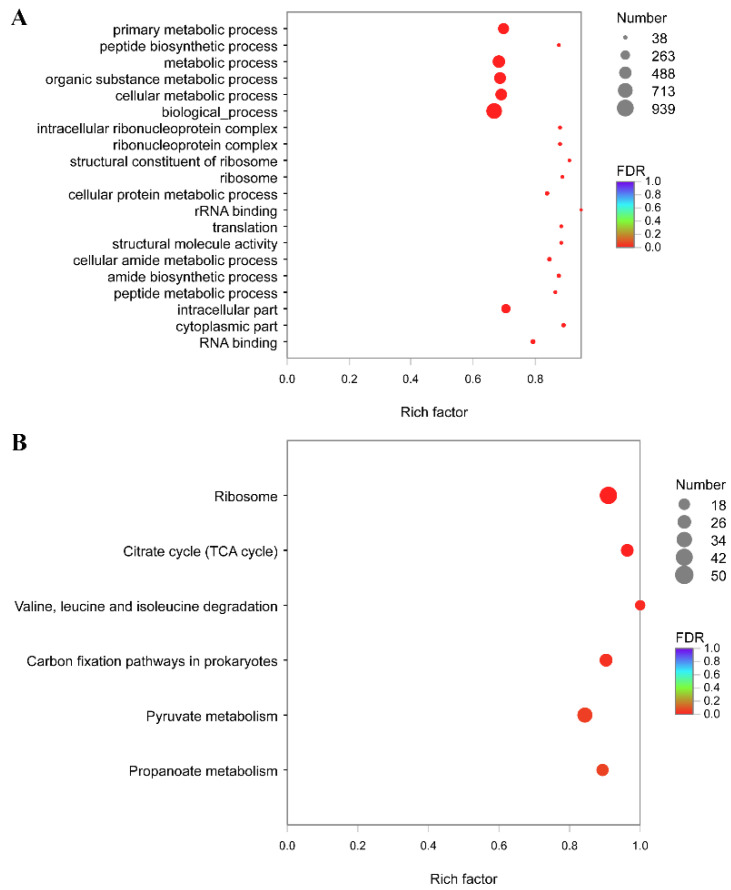
Analysis of differentially expressed genes between 18 and 28 °C. (**A**) GO terms of DEGs between 18 and 28 °C were classified based on their molecular functions, involvement of biological processes and cellular components. The size of the dot indicates the number of genes/transcripts in this GO Term, and the color of the dot corresponds to different FDR ranges. (**B**) KEGG enrichment of differentially expressed genes between 18 and 28 °C. The size of the dot indicates the number of genes in this pathway, and the color of the dot corresponds to different q-value ranges.

GO enrichment on 2590 differentially expressed genes at 28 °C compared to that at 18 °C showed that several processes were significantly enriched, such as “primary metabolic process”, “peptide biosynthetic process”, and “intracellular ribonucleoprotein complex” (Figure 4A). We also mapped the 2590 DEGs to the reference canonical pathway in KEGG (Figure 4B). “Ribosome”, “TCA cycle”, “Carbon fixation pathways in prokaryotes”, and “Valine, leucine, and isoleucine degradation” were the most significant enriched pathways of DEGs. Equally representative pathways were “Pyruvate metabolism” and “Propanoate metabolism”.

**Figure 5:**
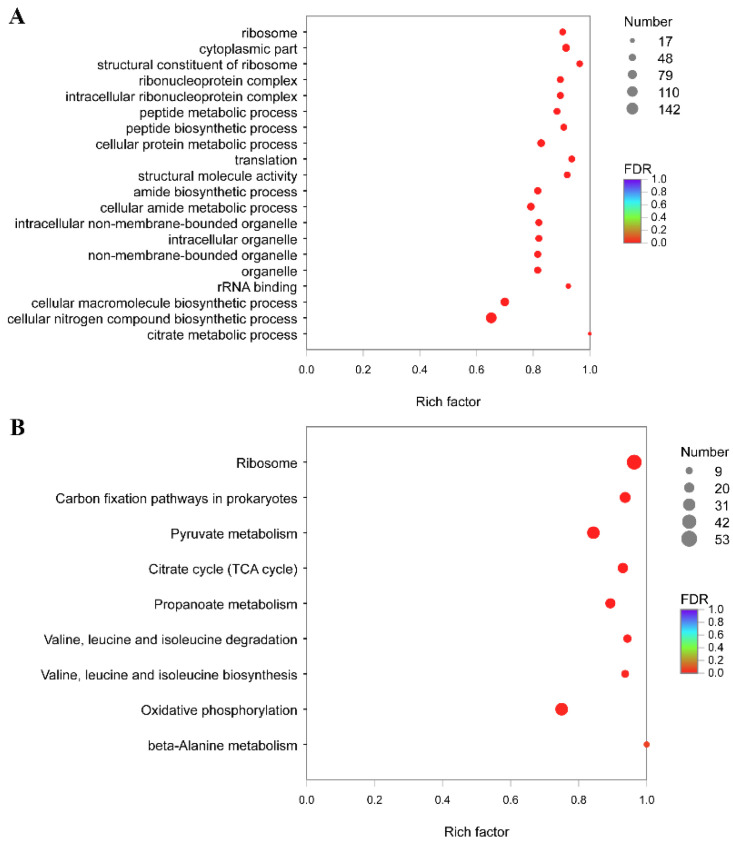
Analysis of differentially expressed genes between 18 and 37 °C. (**A**) GO terms of DEGs between 18 and 37 °C were classified based on their molecular functions, involvement of biological processes and cellular components. The size of the dot indicates the number of genes/transcripts in this GO term, and the color of the dot corresponds to different FDR ranges. (**B**) KEGG enrichment of differentially expressed genes between 18 and 37 °C. The size of the dot indicates the number of genes in this pathway, and the color of the dot corresponds to different q-value ranges.

GO enrichment on 2021 differentially expressed genes at 37 °C compared to that at 18 °C showed that several processes were significantly enriched, such as “ribosome”, “cytoplasmic part”, and “peptide biosynthetic process” (Figure 5A). We also mapped the 2021 DEGs to the reference canonical pathway in KEGG (Figure 5B). “Ribosome”, “TCA cycle”, “Carbon fixation pathways in prokaryotes”, and “Propanoate metabolism” were the most significant enriched pathways of DEGs. Equally representative pathways were “Valine, leucine, and isoleucine degradation” and “Valine, leucine, and isoleucine biosynthesis”.

The Venn diagram compared the genes significantly changed under different temperatures and showed the specifically differentially expressed genes: compared to the 18 °C-treated group, 110 genes were specifically down-regulated under 37 °C, 175 genes were specifically up-regulated under 37 °C, 418 genes were specifically down-regulated under 28 °C, and 45 genes were specifically up-regulated under 28 °C (Figure S4B). These genes were later chosen for further functional annotation and enrichment analysis, in order to investigate the mechanisms underlying the mesophilic growth ability and virulence regulation of *A*. *salmonicida* SRW-OG1.

### 3.5. Functional Annotation and Enrichment Analysis of Genes Specifically Down-Regulated under 28 °C

We mapped 418 genes specifically downregulated at 28 °C to the reference canonical pathway in KEGG (Figure S5A). “Translation” and “Amino acid metabolism” were the most representative pathways of DEG. Equally representative pathways were “Metabolism of cofactors and vitamins” and “Membrane transport”. Among the enriched KEGG pathways, there were some known pathways closely related to environmental adaptation and virulence regulation, for example: “Environmental adaptation”, “Drug resistance: antimicrobial”, and “Cell growth and death”. KEGG enrichment was also carried out on the 418 DEGs, and it was worth noting that the pathway of “ABC transporters” showed significant enrichment of DEGs (Figure S5B).

### 3.6. Functional Annotation and Enrichment Analysis of Genes Specifically Up-Regulated under 28 °C

The 45 genes specifically up-regulated under 28 °C were mapped to reference canonical pathways in KEGG (Figure S6A). Pathways that had the largest representation by DEGs were “Signal transduction”, “Carbohydrate metabolism”, “Cellular community-prokaryotes”, “Amino acid metabolism”, and “Cell motility”. Some of the pathways were associated with environmental adaptation and virulence regulation, for example: “Drug resistance: antimicrobial”, “Cellular community-prokaryotes”, “Cell growth and death”, and “Cell motility”. KEGG enrichment was also carried out on the 345 DEGs, and it was worth noting that the pathway of “Two-component system” and “Bacterial chemotaxis” showed significant enrichment of DEGs (Figure S6B).

### 3.7. Functional Annotation and Enrichment Analysis of Genes Specifically Down-Regulated under 37 °C

The 110 genes specifically down-regulated under 37 °C were mapped to reference canonical pathways in KEGG (Figure S7A). Pathways with the largest representation by DEGs were “Energy metabolism”, “Metabolism of cofactors and vitamins”, “Infectious disease: bacterial”, “Translation”, “Metabolism of other amino acids”, “Folding, sorting, and degradation”, and “Amino acid metabolism”. Some pathways were associated with environmental adaptation and virulence regulation, for example: “Drug resistance: antimicrobial”, “Cell growth and death”, “Cellular community-prokaryotes”, and “Environmental adaptation”. KEGG enrichment was also carried out on the 961 DEGs, and it was worth noting that the pathway of “Plant-pathogen interaction” and “Salmonella infection” showed significant enrichment of DEGs (Figure S7B).

### 3.8. Functional Annotation and Enrichment Analysis of Genes Specifically Up-Regulated under 37 °C

The 175 genes specifically down-regulated under 37 °C were mapped to reference canonical pathways in KEGG (Figure S8A). Pathways with the largest representation by DEGs were “Carbohydrate metabolism”, “Amino acid metabolism”, “Metabolism of cofactors and vitamins”, “Membrane transport”, “Cellular community-prokaryotes”, and “Drug resistance: antimicrobial”. Some pathways were associated with environmental adaptation and virulence regulation, for example: “Drug resistance: antimicrobial”, “Infectious disease: bacterial”, and “Cell motility”. KEGG enrichment was also carried out on the 1060 DEGs, and it was worth noting that the pathway of “Drug metabolism”, “Cationic antimicrobial peptide resistance”, “Pathogenic *E*. *coli* infection”, and “ABC transporters” showed significant enrichment of DEGs (Figure S8B).

### 3.9. The Sequence Alignment Analysis of aerA and hlyA of A. salmonicida

According to the genetic distance of *aerA* and *hlyA* calculated by MEGA.7 and the evolutionary tree (Figures S9–S12), *A*. *salmonicida* SRW-OG1 was the closest to *A*. *dhakensis* (strain: 71431) and *A*. *hydrophila* (strain: OnP3.1). The amino acid sequence alignment results of aerolysin and hemolysin between *A*. *salmonicida* SRW-OG1 and other pathogenic bacteria also showed that the amino acid sequence similarity of aerolysin and hemolysin was the highest compared with *A*. *dhakensis* (strain: 71431) and *A*. *hydrophila* (strain: OnP3.1) (Figures S10 and S12).

### 3.10. Protein Structure Prediction of Aerolysin and Hemolysin of A. salmonicida

**Figure 6:**
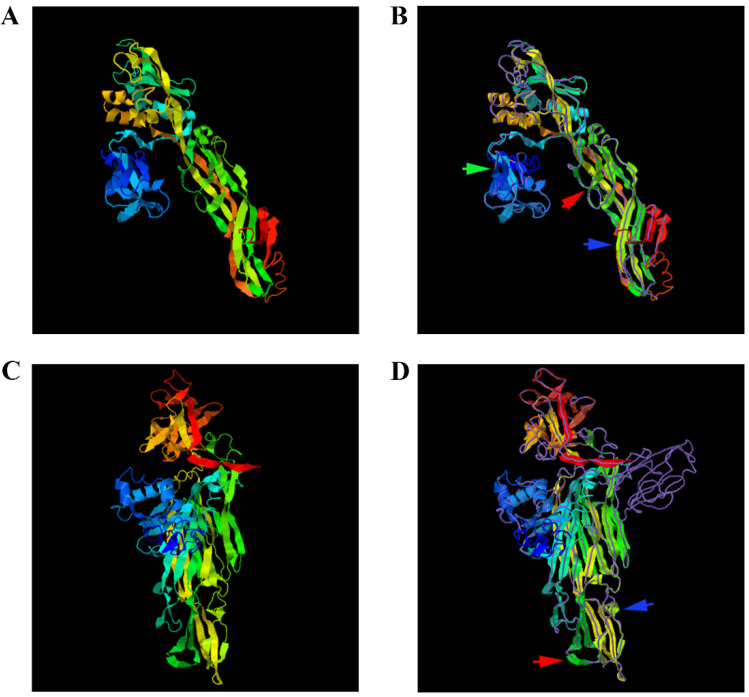
Protein structure prediction of AerA and HlyA of *A*. *salmonicida***.** (**A**) shows the predicted *A*. *salmonicida* AerA protein structure model; (**B**) is the structural comparison of the aerolysin domain of *A*. *salmonicida* (color) and the closest known protein structural model (purple) in PDB library (PDB ID: 1z52A). Aerolysin protein is composed of two lobes: a small lobe (pointed out by the green arrow) and a large lobe (pointed out by the blue arrow). The proaerolysin TMD is pointed out by the red arrow. Domain 1 of the small lobe and domain 2 of the large lobe have high affinity for ankyrin. (**C**) shows the predicted *A*. *salmonicida* HlyA protein structure model; (**D**) is the structural comparison of the aerolysin domain of *A*. *salmonicida* (color) and the closest known protein structural model (purple) in PDB library (PDB ID: 1xezA). *A*. *salmonicida* hemolysin protein is composed of three different domains, and the hemolysin domain (pointed out by the red arrow) is the core of its structure and function. The rim domain loop (pointed out by the blue arrow) in the hemolysin domain is the key for the toxin to bind to erythrocyte membranes and lipids with phosphocholine.

The protein structures of aerolysin and hemolysin were predicted by I-TASSER: according to the cluster ranking size, among the top five structural clusters, we selected the models with the highest confidence (Figure 6). We used the TM-align structure alignment program to match the models to all structures in the PDB library and took the highest TM-score to screen out the protein with the closest similarity. Therefore, the proteins we selected with the closest structural similarity to the predicted protein model in PDB were PDB ID: 1z52A (Figure 6A,B) and PDB ID: 1xezA (Figure 6C,D).

The *A*. *salmonicida* aerolysin protein crystal structure model closest to the known protein structural model in the PDB library was the proaerolysin from *A*. *hydrophila*. The structure of *A*. *hydrophila* proaerolysin consists of two lobes, the amino-terminal domain 1 and the less stable carboxy-terminal domain, which are divided into domains 2–4. Aerolysin is secreted as a soluble precursor called proaerolysin and requires high-affinity interaction with glycosyl-phosphatidylinositol (GPI)-anchored proteins on the plasma membrane of target cells, promoting the formation of stable amphiphilic heptamers. The heptamer can insert into the membrane and form channels that eventually lead to cell death [37]. Domain 1 is essential for high-affinity binding of receptor proteins. Of course, the role of domains 2 to 4 is also indispensable. The existence of some aromatic residues confirms that the existence of domains 1 and 2 lead to proaerolysin to bind the high overall affinity for receptors [38]. The results of I-TASSER comparison of *A*. *hydrophila* proaerolysin domain and the proaerolysin template in the PDB library showed that they had very high similarity.

The *A*. *salmonicida* hemolysin protein crystal structure model closest to the known protein structural model in the PDB library was the *Vibrio cholerae* HlyA pro-toxin. The V. cholerae HlyA pro-toxin consists of four distinct domains distributed in a cruciform shape, with three spherical domains of the same size connected together to a central scaffold provided by the cytolysin domain, the periphery of the whole cruciform structure contains three α-helices and multiple β-sheet strands. The *V*. *cholerae* HlyA pro-toxin has three completely unique domains, within the context of pore-forming toxins, projecting outward from the cytolytic core [39]. The cytolysin domain is the structural and functional core of the *V*. *cholerae* HlyA pro-toxin, and it contains two subdomains, similar to the β-sandwich and rim domains of the Staphylococcus aureus toxin. However, this functionally similar rim domain loop is critical for toxin binding to erythrocyte membranes and lipids with phosphocholine headgroups [40]. According to the comparison results of I-TASSER, the structure of *A*. *salmonicida* HlyA protein also has 4 different domains, which are distributed in a cross shape in space. Therefore, the structure of aerolysin and hemolysin were highly conserved, which indicated their close relationship with cellular toxicity and virulence.

### 3.11. Enhanced Hemolytic Activity of Mesophilic A. salmonicida SRW-OG1 Was Induced by Elevated Temperature

**Figure 7:**
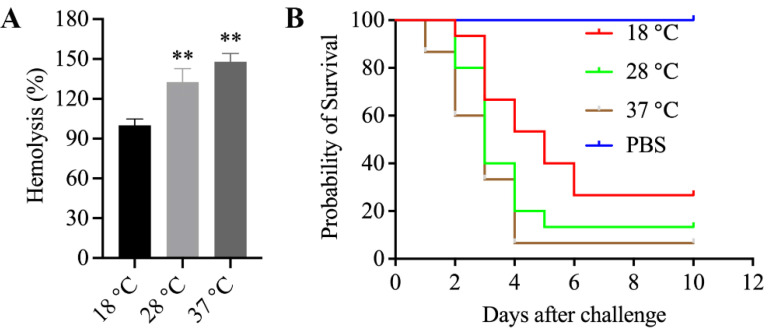
Hemolytic activity and toxicity of ECPs isolated from *A*. *salmonicida* cultured under different temperatures. (**A**) shows the hemolysis (%) histogram of *A*. *salmonicida* ECPs from different culture temperatures; (**B**) is the number of *E*. *coioides* that survived after injection with ECPs of *A*. *salmonicida* from different culture temperatures. Data are presented as mean ± standard deviation (SD) (*n* = 3). ** *p* < 0.01.

**Figure 8:**
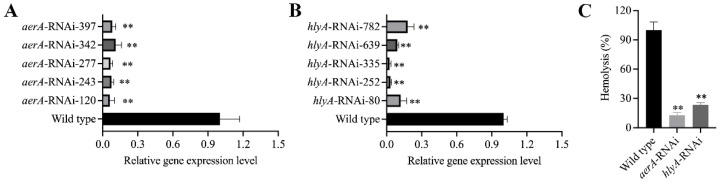
Effects of *aerA* and *hlyA* on the hemolytic activity and virulence of *A*. *salmonicida*. (**A**,**B**) To characterize effects of *aerA* and *hlyA*, five *aerA*- and *hlyA*-RNAi strains were constructed, and the silencing efficiencies were validated by qRT-PCR; (**C**) shows the hemolysis (%) histogram of wild-type, *aerA*-RNAi, and *hlyA*-RNAi *A*. *salmonicida*. Data are presented as mean ± standard deviation (SD) (*n* = 3). ** *p* < 0.01.

As we have mentioned above, the increased expression of *aerA* and *hlyA* at high temperature was confirmed by RNA-seq and qRT-PCR. Since aerolysin and hemolysin are the most well-known and -characterized virulence factors of pathogenic *Aeromonas* strains, the relationship between them and mesophilic virulence was worth exploring. Therefore, we cultured the *A*. *salmonicida* at 18, 28, and 37 °C, and the ECPs were isolated to carry out the hemolytic activity assay and artificial infection. Our results showed that the ECPs from high-temperature-cultured *A*. *salmonicida* displayed enhanced hemolytic activity (Figure 7A) and virulence (Figure 7B). We also constructed the *aerA* and *hlyA* silencing strains (Figure 8A,B) in order to further validate the effects of *aerA* and *hlyA* on the hemolytic activity and virulence of *A*. *salmonicida*. Our results showed that the hemolytic activity of *A*. *salmonicida* was significantly impaired in the *aerA* and *hlyA* silencing strains (Figure 8C). The LD_50_ value of wild-type, *aerA*-RNAi, and *hlyA*-RNAi strains were 2.1 × 10^5^ cfu/mL, 3.4 × 10^8^ cfu/mL, and 1.6 × 10^7^ cfu/mL, respectively. The virulence of *A*. *salmonicida* were significantly impaired in the *aerA* and *hlyA* silencing strains. These results proved that enhanced hemolytic activity of mesophilic *A*. *salmonicida* SRW-OG1 was induced by elevated temperature.

## 4. Discussion

*A*. *salmonicida* is a low-temperature pathogen that can cause furunculosis outbreaks in a variety of fish [41]. In recent years, some *A*. *salmonicida* strains have been identified as mesophilic and demonstrated to be capable of infecting humans as well as mammals [42,43,44]. In the present study, transcriptomic analysis of the SRW-OG1 firstly isolated from the *E*. *coioides* was performed at different temperatures, and 2784 temperature-dependent differentially expressed genes were identified in its transcriptome. These differentially expressed genes are candidate factors to explain the mesophilicity and high-temperature pathogenicity of this strain.

Based on the KEGG pathway enrichment analysis on all of the DEGs, we found that most of the affected pathways were related to cellular metabolism (Figure 3B). As we know, water temperature is the most important environmental factor affecting bacteria-induced disease in aquaculture, while cellular metabolism is one of the biological processes that are widely affected by temperature and closely related to bacterial pathogenicity, and can be used as an interpretation perspective, as well as a monitoring biomarker of bacterial pathogenicity changes [26]. For instance, water temperature is considered to be the most important environmental factor affecting the *Pseudomonas plecoglossicida* induced “white spot disease” [45]. To determine the *P*. *plecoglossicida* global metabolic responses to temperature variation, analysis with GC/MS and RNA-seq is carried out. The results indicate that substance accumulation and energy consumption for survival of pathogens in macrophages and colonizing hosts are accompanied by changes of related virulence gene expression at virulent temperature. These metabolic adjustments assist *P*. *plecoglossicida* to survive in adverse environments, proliferate in the host, colonize, and resist host immune clearance in the initial stages of infection [45]. In the present study, “TCA cycle” and “Valine, leucine, and isoleucine degradation” were the most significant enriched pathways of all DEGs (Figure 3B). Equally representative pathways were “Pyruvate metabolism” and “Valine, leucine, and isoleucine biosynthesis”. The TCA pathway is a very important part of the central metabolic pathway, which supplies precursors for biosynthesis and is the source for energy in bacteria [46]. An impaired TCA cycle was associated with a decrease in the virulence of pathogenic bacteria [47]. Huang et al. [48] proved that the TCA cycle is closely related to the adhesion and virulence of *Vibrio alginolyticus*, while it is sensitive to the change of temperature. Pyruvate and its metabolism are crucial for the fitness and virulence for several bacterial pathogens, including *Borrelia burgdorferi*, *Leptospira interrogans*, *Listeria monocytogenes*, *Vibrio parahaemolyticus*, *Yersinia pseudotuberculosis*, *Staphylococcus aureus*, and uropathogenic *E*. *coli* [49,50,51,52,53,54]. For example, the pyruvate–TCA cycle node as a focal point for controlling the host colonization and virulence of *Yersinia* [55]. Lamia et al. identify pyruvate as a novel regulatory signal for the coordination of the *S*. *aureus* virulon through intricate regulatory activities [55]. The metabolism of pyruvate is sensitively regulated by temperature in pathogenetic bacteria, such as *P*. *plecoglossicida* [55]. The concentration of pyruvate in *P*. *plecoglossicida* is significantly different between virulent and avirulent temperatures [55]. Additionally, the integrated analysis of RNA-seq and iTRAQ revealed the modulation of pyruvate metabolism, which contribute to *V*. *parahaemolyticus* cold tolerance [55]. Valine, leucine, and isoleucine are required for protein synthesis and serve as precursors of branched-chain fatty acids, the major fatty acids of bacteria [55]. The valine, leucine, and isoleucine metabolism has been proved to be crucial for adaptation to environmental starvation and virulence in pathogens such as *Aspergillus fumigatus* [56] and *Bacillus anthracis* [57]. For example, Kaiser et al. prove the valine, leucine, and isoleucine metabolism is closely related to the growth, environmental adaptation, and virulence of *S*. *aureus* [55]. Orasch et al. find that the leucine metabolism is crucial for adaptation to iron starvation and virulence in *A*. *fumigatus* [56]. Dutta et al. report the influence of valine, leucine, and isoleucine metabolism on *B*. *anthracis* growth and virulence [57]. The relationship between temperature and the valine, leucine, as well as isoleucine metabolism in pathogenic bacteria has also been reported. For example, Tsuda et al. prove that the valine, leucine, and isoleucine metabolism play important roles in the cold tolerance of the genus *Sporosarcina* and that these bacteria modulate their fatty acid compositions in response to the growth environment [58]. Zhu et al. find that the valine, leucine, and isoleucine metabolism are required for low-temperature growth of *L*. *monocytogenes* [59]. Therefore, based on these previous research reports, we can easily find that the virulence, temperature, and cellular metabolism are closely related in bacteria, which is consistent with the findings of this study.

According to the KEGG pathway enrichment analysis on all of the DEGs, there were 105 genes enriched in “ABC transporters” (Figure 3B), such as *znuABC*, *oppABCDF*, and *afuABC*. Disease-causing bacteria depend on the acquisition of a diverse set of nutrients from their hosts to engage in successful pathogenesis. For example, Manganese (Mn), zinc (Zn), and iron (Fe), as well as other transition metals, are vital to living beings, because they play important roles in protein structure and function [60]. Pathogenic microorganisms must obtain these micronutrients from the host, while the latter attempts to intercept them through a process called nutritional immunity [61,62,63,64]. As the second most-abundant transition metal in the majority of organisms, Zn has both catalytic and structural functions in proteins [65]. Indeed, Zn-binding proteins account for approximately 4 to 8% of all proteins produced by prokaryote organisms [66]. In some bacterial pathogens, the lack of a high-affinity Zn transport system leads to reduced virulence [63]. In the light of these important roles of Zn in bacterial physiology, it is not surprising that Zn sequestration represents an essential innate defense strategy. A typical example of Zn restriction is the staphylococcal abscess, which lacks Zn [67,68]. In bacteria, Zn import through the plasma membrane is mainly promoted by the ZnuABC [69]. The importance of Zn transporters to overcoming nutritional immunity and calprotectin has been demonstrated for several pathogens. For example, *Pseudomonas aeruginosa* relies on the *znuABC* Zn transporter to overcome calprotectin-mediated growth inhibition [70]. Recent studies have shown that the ZnuABC Zn uptake system of *S*. *typhimurium* is necessary for resistance to Zn chelation mediated by calprotectin accumulation in the intestine after infection [71]. In addition, *S*. *typhimurium* uses calprotectin-mediated Zn chelation to compete with the host microbiota, which are not well adapted to nutritional deficiency [71]. In recent studies, the importance of *znuABC* was revealed by investigating how *P*. *plecoglossicida* responds to Zn nutritional immunity during infection. While diverse Zn-sequestration strategies against *P*. *plecoglossicida* have been observed in *E*. *coioides*, a common denominator of all these strategies is pushing *P*. *plecoglossicida* out of its ideal environment [36,72].

The role of OppABCDF importers is to capture peptides from the extracellular environment to serve as sources of plasma carbon and nitrogen [73]. In addition to their nutritional role, Opp systems have also been associated with virulence in several bacteria. Studies performed on pathogenic bacteria of the genera *Staphylococcus* sp., *Streptococcus* sp., and *Mycobacterium* sp. have shown that Opp mutant strains show reduced virulence [74]. Resent research on *Vibrios* also showed that Opp systems are essential for in vitro hemolytic activity and biofilm production [75]. The peptides captured by Opp systems can be used as signaling molecules in intercellular communication, which allows the bacteria to coordinate the expression of specific genes at a population level. The control of virulence has been linked to communication via signal peptides [76]. For example, the peptides could also activate a pleiotropic virulence regulon, as demonstrated in the case of *Bacillus thuringiensis* [77], or by stimulating adherence of pathogenic streptococci to human cells [76]. Liu et al. [78] find that *oppABCDF* is closely related to the adhesion and virulence of *V*. *alginolyticus*, and sensitively regulated by temperature.

AfuABC binds and transports a particular set of phosphorylated carbohydrates, which is conserved across bacteria, including a large number of Gram-negative pathogens such as *Vibrio cholerae*, *Haemophilus influenzae,* and *Citrobacter rodentium*. AfuABC can mediate sugar–phosphate transport into bacteria and *C*. *rodentium* lacking AfuA are significantly impaired in competitive growth within the mammalian gut, indicating that AfuABC-based recognition and uptake of sugar–phosphates within the intestinal lumen plays a critical role during infection [79].

Our results of the KEGG pathway enrichment analysis on all of the DEGs showed that there were 51 genes enriched in “Bacterial chemotaxis” (Figure 3B), such as *mcp*, *fliG*, *motA*, *cheB*, *cheY*, and *cheV*. During the pathogenic process of bacteria, attachment to external surfaces of a host is an important initial step in colonization and subsequent occurrence of infection [80]. The relation between chemotaxis and adhesion has been extensively studied. For example, chemotaxis of cholera vibrios is proved to facilitate the association of these bacteria with the mucosal surface [81]; chemotaxis of Vibrio furnissii is proved to catalyzes the first step in colonizing chitin [82]; chemotaxis was also proved to play key roles in the colonization of adherent invasive *E*. *coli*. Therefore, more and more evidence has shown that the chemotaxis pathway might play a key role in the adhesion of various pathogens. Huang et al. [80] find that the bacterial chemotaxis pathway is closely related to the adhesion and virulence of *V*. *alginolyticus*, and sensitively regulated by temperature.

Functional annotation and enrichment analysis of genes specifically changed under 28 °C indicated that some functional clusters associated with environmental adaptation and virulence regulation were significantly affected, for example: “Environmental adaptation”, “Two-component system”, “Inorganic ion transport and metabolism”, “Bacterial chemotaxis”, and “ABC transporters” (Figures S5 and S6). “Bacterial chemotaxis”, “Two-component system”, and “ABC transporters” have long been recognized as important regulators of bacterial virulence, as we mentioned above. From the results of this study, they also played an important role in the environmental adaptation and virulence regulation of *A*. *salmonicida* at 28 °C.

Functional annotation and enrichment analysis of genes specifically changed under 37 °C revealed some significantly affected functional clusters, such as “Cell cycle control, cell division, chromosome partitioning”, “Infectious disease: bacterial”, “*Salmonella* infection”, and “Pathogenic *E*. *coli* infection” (Figures S7 and S8). As we know, *Salmonella* and *E*. *coli* are pathogenic to mammals and humans. Thus, on the one hand, the genes enriched in “*Salmonella* infection” and “Pathogenic *E*. *coli* infection” further indicated that SRW-OG1 may infect humans and mammals and needs to be taken seriously. On the other hand, these genes might be one of the keys to pathogenicity of *A*. *salmonicida* at 37 °C.

Except the functional unknown genes, the most significantly affected genes have been reported to be more or less related to bacterial pathogenicity, such as *aerA*, *argF*, *dhaL*, and *aceA*. *aerA* is the encoding gene of aerolysin, which is a kind of *Aeromonas* chief toxin [19]. *argF* is a key member of the arginine biosynthesis pathway, while the *argF* deletion mutant of *Mycobacterium tuberculosis* is rapidly sterilized in mice [83]. *dhaL* is associated with glycerol metabolism, which contributes to the cell fitness and virulence of *Actinotignum schaalii* [84] and *Listeria monocytogenes* [85]. AceA and is the Cu fist binding transcription factor, which senses high levels of Cu, and induces the Cu P-type ATPase CrpA as a detoxification mechanism. AceA is a virulence factor for the human pathogenic *A*. *fumigatus* [86].

We also paid attention to the expression of heat shock proteins and Type III secretion system (T3SS) encoding genes. Heat shock proteins act as chaperones that enable the optimal folding of newly synthesized proteins under stress conditions. Null mutation of heat shock protein affects many physiological processes in pathogenic bacteria, including those that are important for virulence, such as motility, biofilm formation, proteolytic activity, and pigment and biosurfactant production [87]. Our results showed that only *ibpA*, a heat shock protein encoding gene, has a significant change in its expression level. In the 28 °C and 37 °C treatment groups, fold change was reduced by 1.2 and 2.5 folds, respectively. This surprised us, because in most cases, the expression level of heat shock protein increases at high temperatures. T3SS is one of the chief virulence factors of *Aeromonas* [19]. Our results showed that the T3SS genes including *trpD*, *pepB*, *folM*, and *copA* were consistently increased in the 28 °C and 37 °C treatment groups, while *tonB*, *fdhD*, *amgK,* and *qor* were consistently decreased in the 28 °C and 37 °C treatment groups. However, whether and how T3SS was involved in the mesophilic virulence of SRW-OG1 still need further investigation.

Among the differentially expressed genes, *aerA* and *hlyA*, the hemolytic genes encoding aerolysin and hemolysin were found to be significantly up-regulated at 28 and 37 °C. As we know, virulence factors of pathogenic *Aeromonas* strains include proteases, aerolysin, hemolysin, enterotoxins, and acetylcholinesterase [19]. *aerA* and *hlyA* are the encoding genes for aerolysin and hemolysin, respectively. The direct detection of *aerA* and *hlyA* is identified as a reliable approach for identifying potentially pathogenic *Aeromonas* strains [23]. Since aerolysin and hemolysin are the most well-known and -characterized virulence factors of pathogenic *Aeromonas* strains, the relationship between them and mesophilic virulence was worth exploring. Through I-TASSER protein structure comparison results, the crystal structures of *A*. *salmonicida* aerolysin protein and *A*. *hydrophila* proaerolysin have the highest similarity (Figure 6A,B), and the crystal structure of *V*. *Cholerae* HlyA pro-toxin is the most similar to that of *A*. *salmonicida* hemolysin protein (Figure 6C,D). The hemolysin protein structure of *A*. *hydrophila* was divided into two lobes; domain 1 of the lobe and domain 2 of the large lobe had high affinity for ankyrin, which laid the foundation for the formation of channels and eventually killing cells [37]. The cross-shaped protein structure of the HlyA pro-toxin of *V*. *cholerae* subtly highlights the cytolysin domain at the core. The rim domain loop of the cytolysin subdomain is a key step for hemolysin to connect to the erythrocyte membrane and form a channel [39]. Based on the fact that proteins with high structural similarity tend to have similar functions to the target, it is speculated that the mesophilic *A*. *salmonicida* aerolysin protein and hemolysin protein can connect to their respective receptor cell membranes to form channels and lead to cell death. Further study proved that the extracellular products (ECPs) purchased from *A*. *salmonicida* SRW-OG1 cultured at high temperatures (28 and 37 °C) showed elevated hemolytic activity and virulence (Figure 7). Moreover, the silence of *aerA* and *hlyA* led to significantly decreased hemolysis and virulence (Figure 8). In conclusion, our results revealed that the mesophilic virulence of *A*. *salmonicida* SRW-OG1 might be due to the enhanced expression of *aerA* and *hlyA* induced by elevated temperature.

Taken together, the *A*. *salmonicida* SRW-OG1 was transcriptomics analyzed at different cultured temperatures in the present study. Through this, several virulence- and environmental-adaptation-related genes were found to be significantly affected by cultured temperatures. This is of great value for further elucidation of the pathogenicity of SRW-OG1 at high temperature. Our work will provide a theoretical reference for the prevention and treatment of aquaculture diseases caused by *A*. *salmonicida* under different water temperatures. However, further research and exploration to clarify the molecular mechanism of temperature-mediated expression of *aerA*, *hlyA,* and other virulence genes is still the direction in which we must focus.

## Data Availability

Read data of RNA-seq were deposited in the GSA database (PRJCA005431).

## Supplementary Materials

The following supporting information can be downloaded at: https://www.mdpi.com/article/10.3390/microorganisms10102033/s1, Table S1: Sequences of primers used in this study; Table S2: Statistics of quality control data; Table S3: DEGs annotation. Figure S1: The Venn diagram compares the genes hit on the NR, Swiss-Prot, COG, and KEGG databases. Figure S2: Distributions of the KEGG annotations. Figure S3: Distributions of the GO annotations. Figure S4: Analysis of differentially expressed genes. Figure S5: Functional annotation and enrichment analysis of genes specifically down-regulated under 28 °C. Figure S6: Functional annotation and enrichment analysis of genes specifically up-regulated under 28 °C. Figure S7: Functional annotation and enrichment analysis of genes specifically down-regulated under 37 °C. Figure S8: Functional annotation and enrichment analysis of genes specifically up-regulated under 37 °C. Figure S9: The sequence analysis of AerA of *A. salmonicida*. Figure S10: The phylogenetic tree of AerA of *A. salmonicida*. Figure S11: The sequence analysis of HlyA of *A. salmonicida*. Figure S12: The phylogenetic tree of HlyA of *A. salmonicida*.
