# Supplementary material for: Enhanced Hemolytic Activity of Mesophilic Aeromonas salmonicida SRW-OG1 Is Brought about by Elevated Temperatures
Source: Microorganisms. 2022 Oct 14;10(10):2033. doi: 10.3390/microorganisms10102033 (PMC9609485; doi:10.3390/microorganisms10102033)
Supplement: Supplementary file 1 [file microorganisms-10-02033-s001.zip › microorganisms-1875019-supplementary/Supplemental Materials/Table S1. Sequences of primers used in this study.pdf]

Table S1. Sequences of primers used in the study

	Sequence (5'->3')	Product size (bp)	Tm (°C)
aerA-F	5'-CCACCTCTTTGTCCCAGTCC-3'	241	59.96
aerA-R	5'-CTGCTGGCCTTGTCCTTGTA-3'		59.96
argF-F	5'-GGTTTTCGGCCTCGTCAAAC-3'	215	60.04
argF-R	5'-GGTGAAACTGATGACCCCGT-3'		59.96
dhaL-F	5'-CCGGATAAAGAAGGTGCCGT-3'	228	60.11
dhaL-R	5'-TGCGAACACATCTTCACCGA-3'		59.97
arcA-F	5'-TCAGAATTCCGTGCCATGCT-3'	215	60.04
arcA-R	5'-TCACCACAGAAACGGTAGCC-3'		59.97
dhaK-F	5'-ATCCGGAGTCGGGGAAGTAA-3'	211	60.03
dhaK-R	5'-ACAACTGATGGGGATGGCAG-3'		60.03
hlyA-F	5'-ACCGGATGGTTCCAATCCAC-3'	247	60.03
hlyA-R	5'-TCCAGCCGCTGAGTTACAAG-3'		60.04
pdhR-F	5'-CGCAGCATAGTAGGCACAGA-3'	231	59.90
pdhR-R	5'-CTCGCCATCCAGTTTCAGGT-3'		60.04
gntU-F	5'-TAGCCAAAATCGGCTCCCAG-3'	217	60.11
gntU-R	5'-TGTCACTGACTGGCTTCGTC-3'		59.97
fruB-F	5'-CTCTGCTGGCAAGCAAACTG-3'	232	60.04
fruB-R	5'-GCGATACCGATGGCCACATA-3'		60.04
yidD-F	5'- TTTCTTCGCTTTGGTTGCGG -3'	226	59.97
yidD-R	5'-CGTTGCAATGGGTAGCTGTC-3'		59.55
aqpZ-F	5'-CATGACCGGCTTCTTCCTGT-3'	220	60.04
aqpZ-R	5'-GATCGGAGCAACCCAGAACA-3'		60.04
aceA-F	5'-TGGCATCGTCCAGGTTCTTC-3'	209	60.04
aceA-R	5'-CTACAAAGTGCAGGCGGGTA-3'		60.04

hppD-F	5'-ATCACCTGACCCACAACGTC-3'	233	59.97
hppD-R	5'-GATCCCCTCCCCCTTGTACT-3'		60.03
fadA-F	5'-TCGAGCTGAACGAGGCATTT-3'	203	60.04
fadA-R	5'-CATGGTGGCAAGACCCAGAT-3'		60.03
yjjW-F	5'-CGGCATCCAGAAAATCGCTG-3'	249	59.97
yjjW-R	5'-ATCTGGCTCACCTCACTTGC-3'		60.04
nirD-F	5'-TGCCAACTCAACGACATCCT-3'	211	59.60
nirD-R	5'-GAAAGCGCTGCTTCTTGAGC-3'		60.45
nirC-F	5'-CTCACCTTCGGGGTGAAGAC-3'	206	60.04
nirC-R	5'-TTCATGAACAGGGTCAGGGC-3'		59.96
treB-F	5'-GCCAGCAGCAGGGAGATAAA-3'	249	60.11
treB-R	5'-GATCCTCGGTATCGTGCTGG-3'		60.04
treC-F	5'-GCTCCTGATAATCCCCCTGC-3'	231	59.96
treC-R	5'-CCATTCTCGGTGCCAAGTCT-3'		60.04
16S-F	5'-AGTACGGCCGCAAGGTTAAA-3'	245	59.96
16S-R	5'-GTGCTGGCAACAAAGGACAG-3'		59.97
gyrB-F	5'-AAGGCCCGTTTCGACAAGAT-3'	219	59.96
gyrB-R	5'-GATGTAGCCACGCTCGATGA-3'		59.97
