# Supplementary figures and images for: Enhanced Hemolytic Activity of Mesophilic Aeromonas salmonicida SRW-OG1 Is Brought about by Elevated Temperatures
Source: Microorganisms. 2022 Oct 14;10(10):2033. doi: 10.3390/microorganisms10102033 (PMC9609485; doi:10.3390/microorganisms10102033)

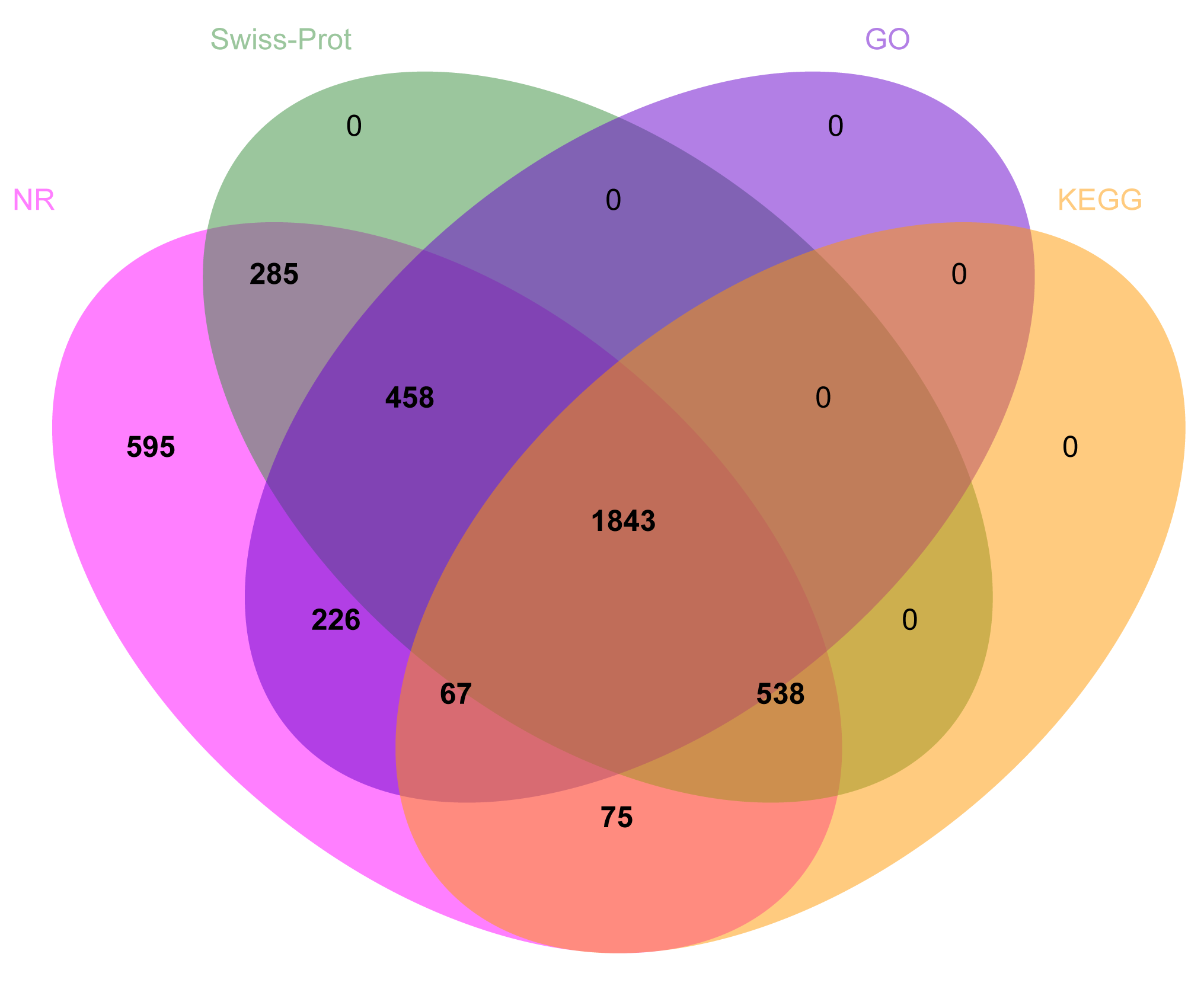

Supplement: Supplementary file 1 [file microorganisms-10-02033-s001.zip › microorganisms-1875019-supplementary/Supplemental Materials/Figure S1.tif]

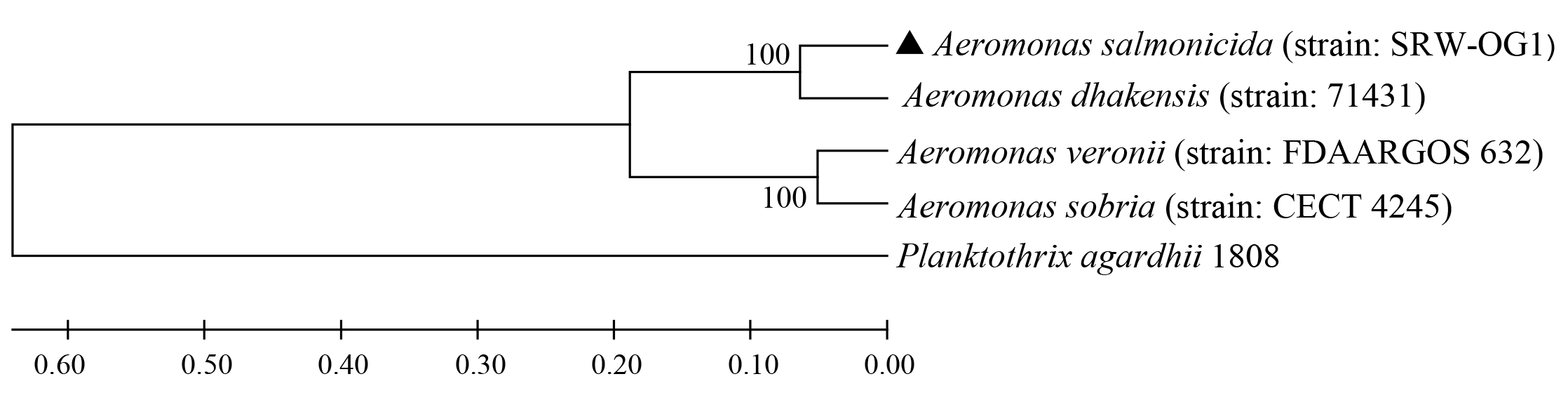

Supplement: Supplementary file 1 [file microorganisms-10-02033-s001.zip › microorganisms-1875019-supplementary/Supplemental Materials/Figure S10.tif]

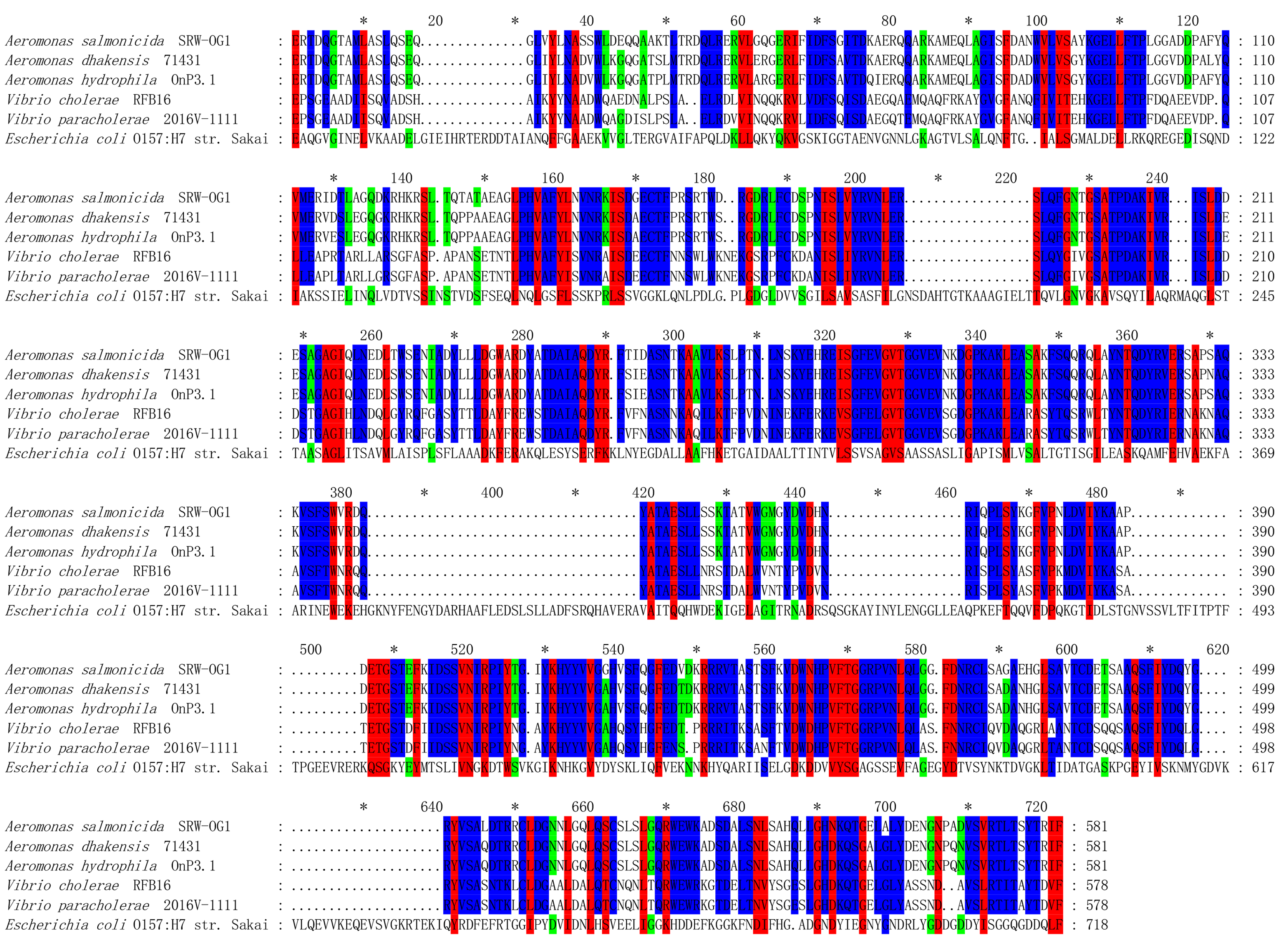

Supplement: Supplementary file 1 [file microorganisms-10-02033-s001.zip › microorganisms-1875019-supplementary/Supplemental Materials/Figure S11.tif]

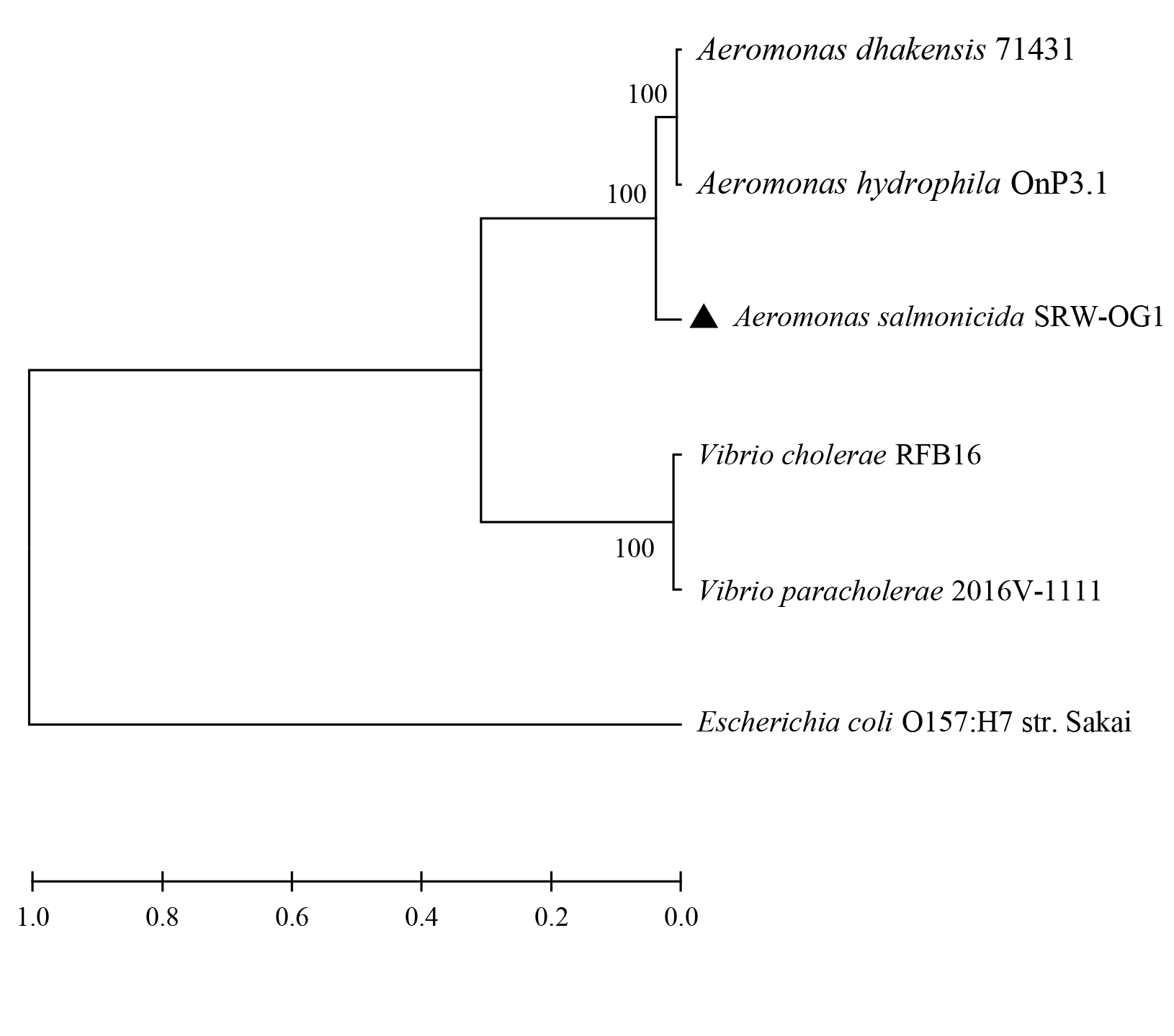

Supplement: Supplementary file 1 [file microorganisms-10-02033-s001.zip › microorganisms-1875019-supplementary/Supplemental Materials/Figure S12.tif]

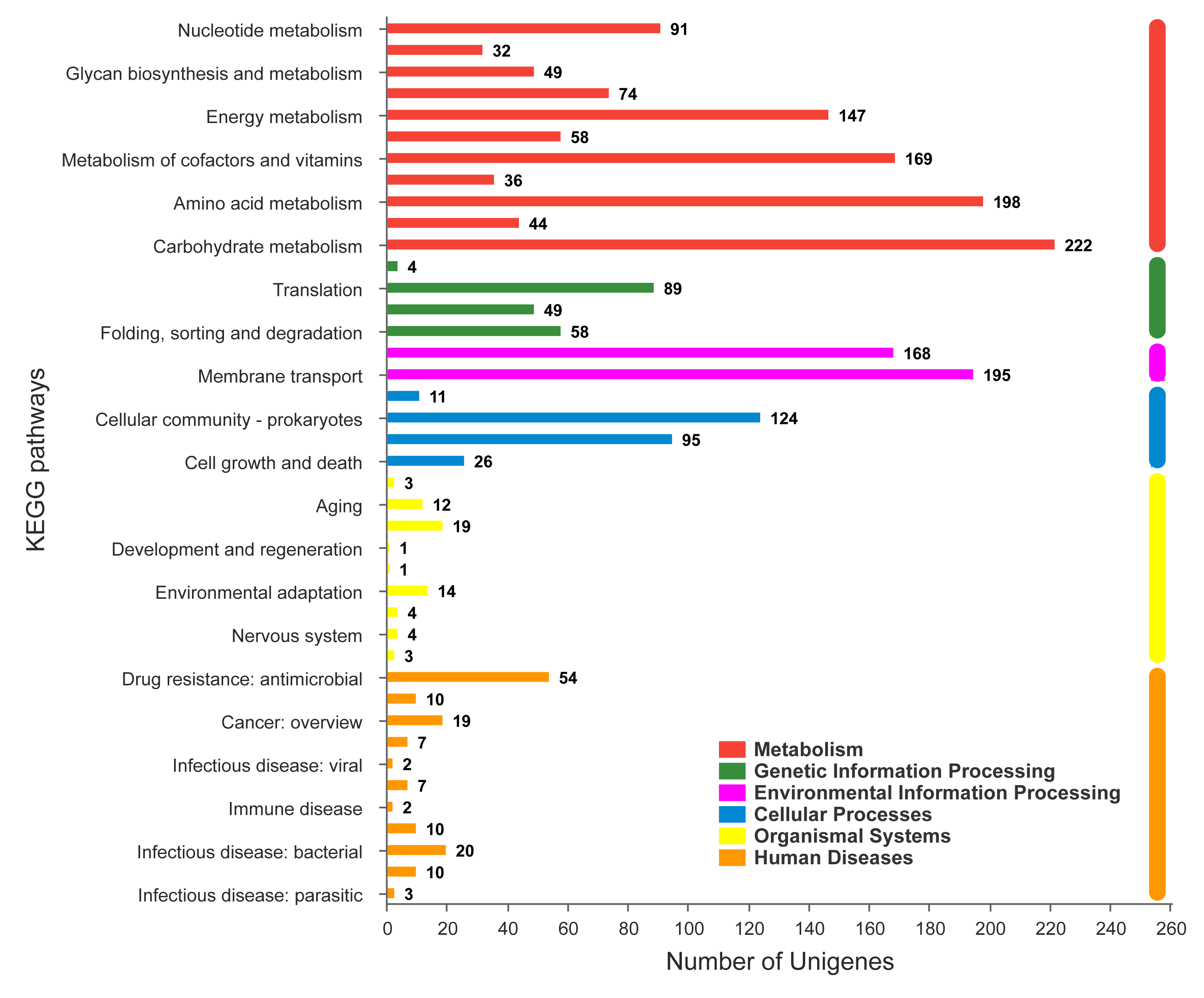

Supplement: Supplementary file 1 [file microorganisms-10-02033-s001.zip › microorganisms-1875019-supplementary/Supplemental Materials/Figure S2.tif]

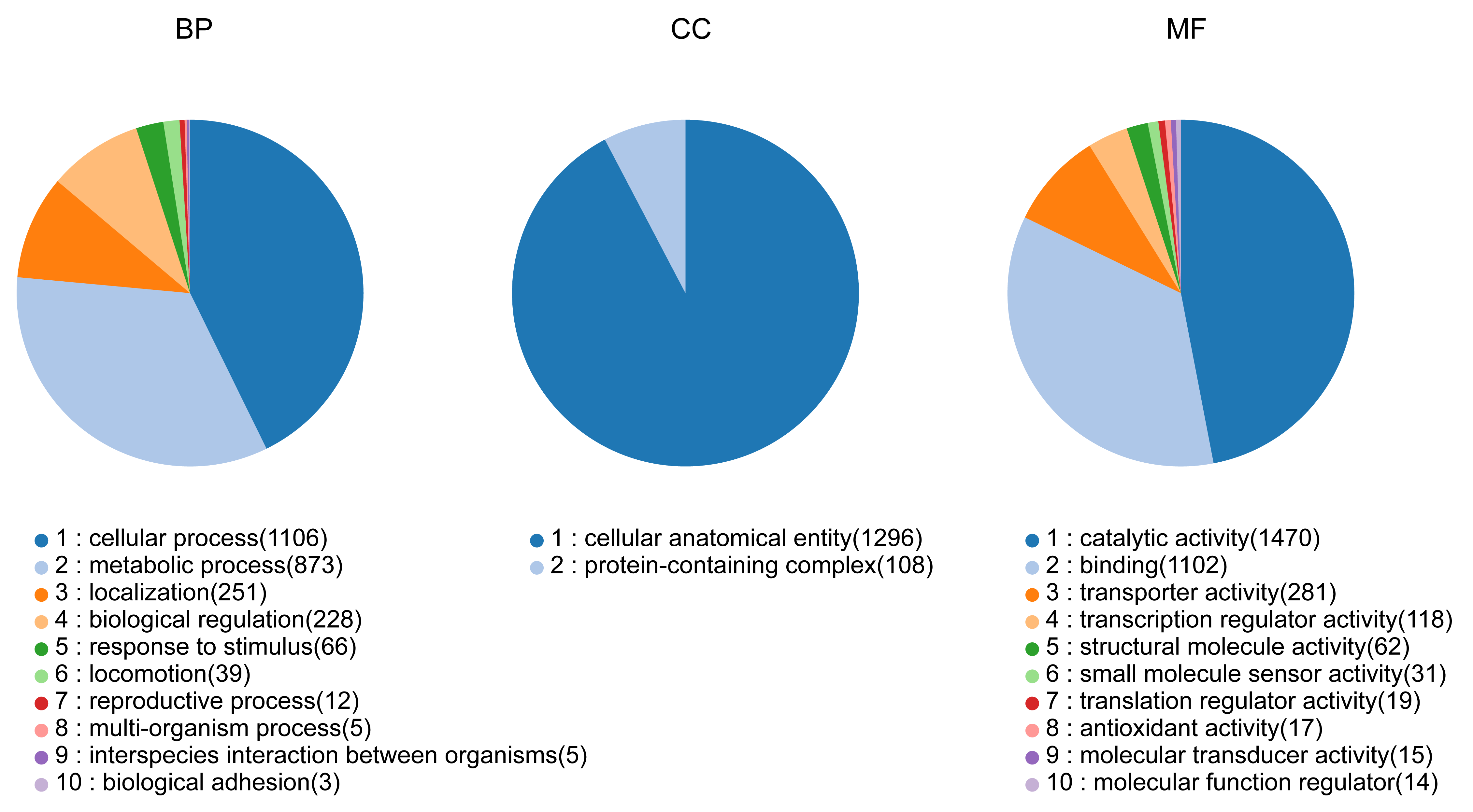

Supplement: Supplementary file 1 [file microorganisms-10-02033-s001.zip › microorganisms-1875019-supplementary/Supplemental Materials/Figure S3.tif]

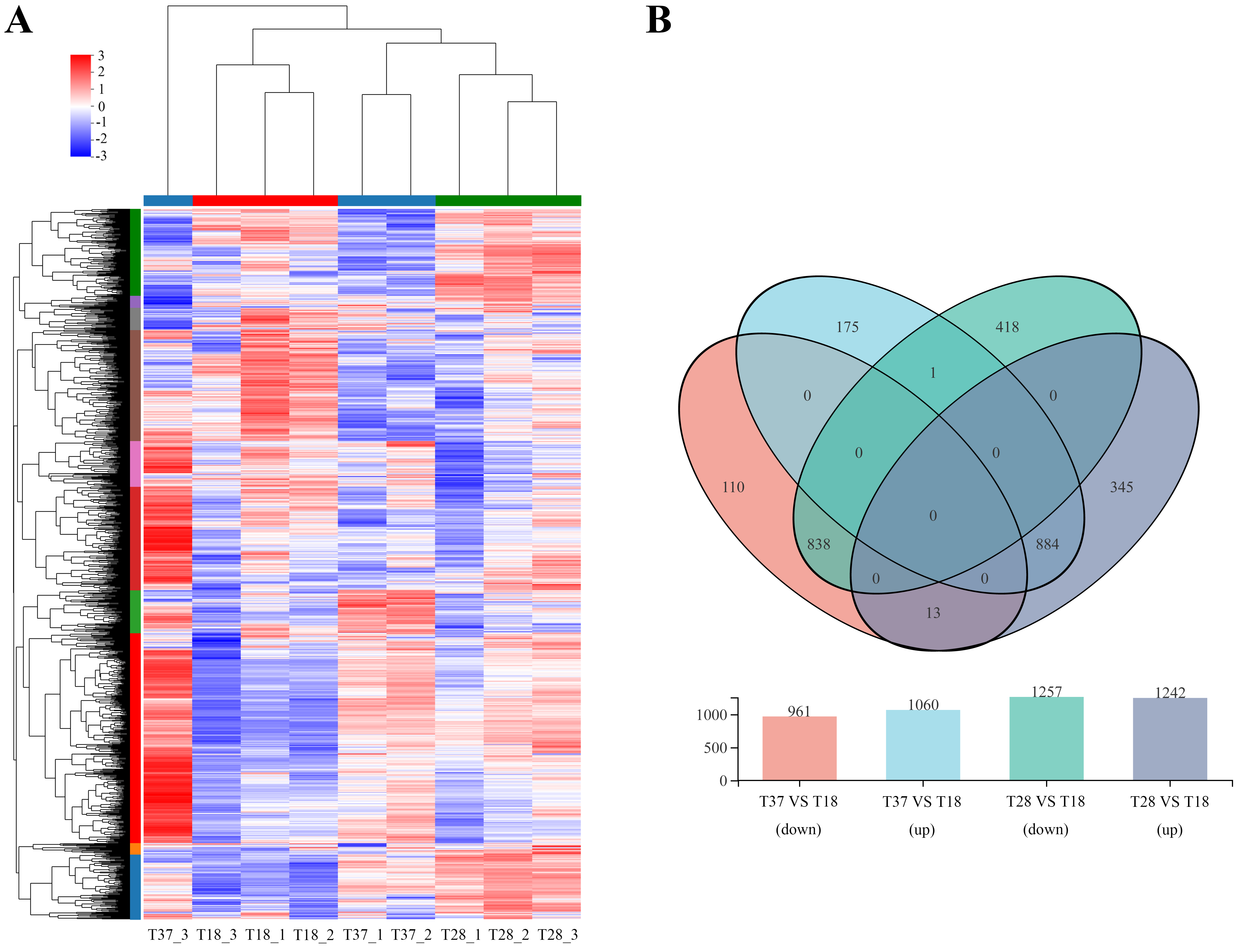

Supplement: Supplementary file 1 [file microorganisms-10-02033-s001.zip › microorganisms-1875019-supplementary/Supplemental Materials/Figure S4.tif]

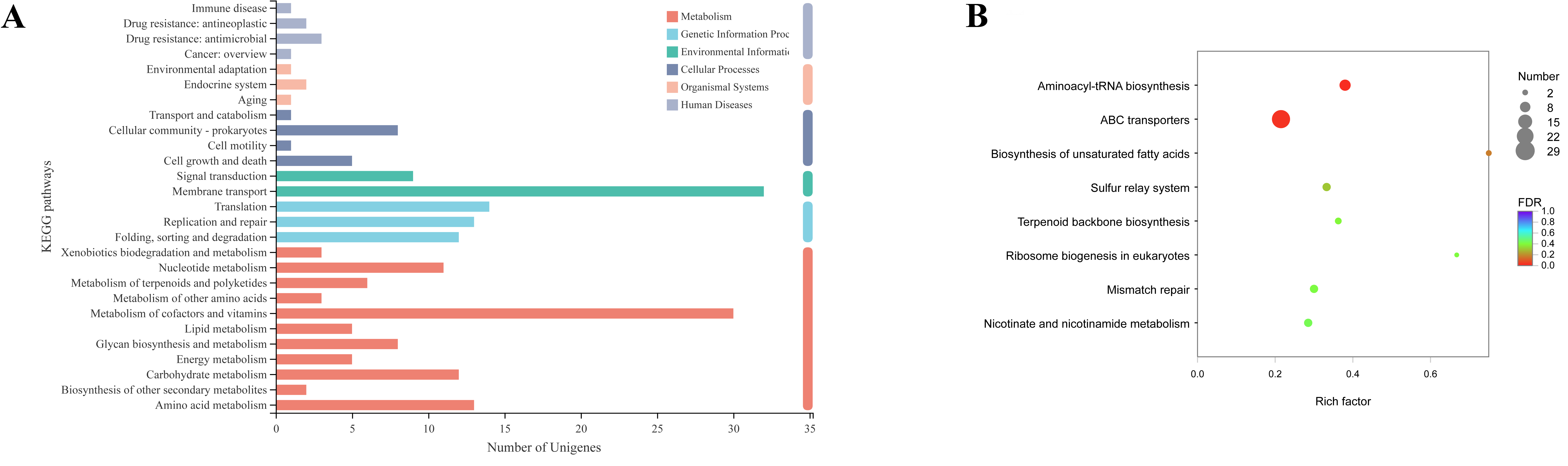

Supplement: Supplementary file 1 [file microorganisms-10-02033-s001.zip › microorganisms-1875019-supplementary/Supplemental Materials/Figure S5.tif]

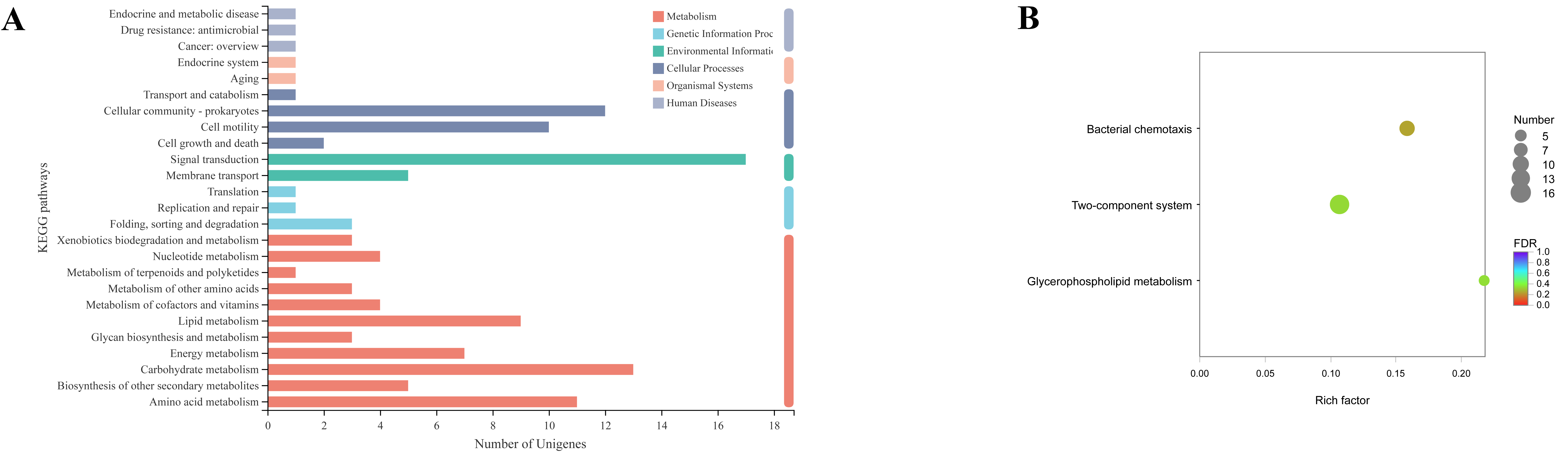

Supplement: Supplementary file 1 [file microorganisms-10-02033-s001.zip › microorganisms-1875019-supplementary/Supplemental Materials/Figure S6.tif]

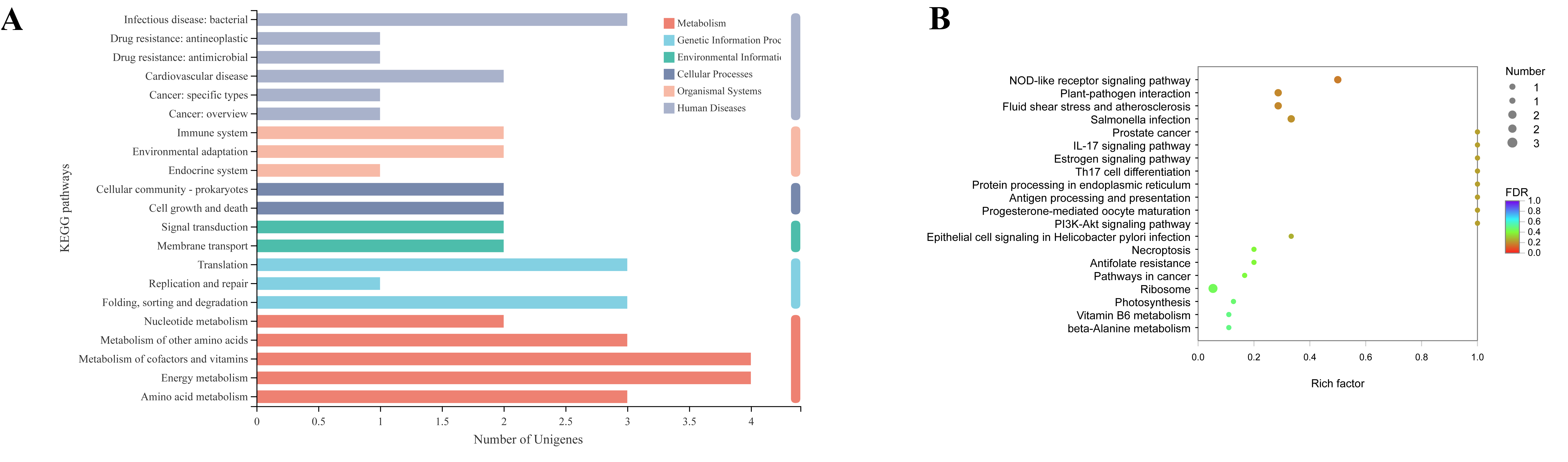

Supplement: Supplementary file 1 [file microorganisms-10-02033-s001.zip › microorganisms-1875019-supplementary/Supplemental Materials/Figure S7.tif]

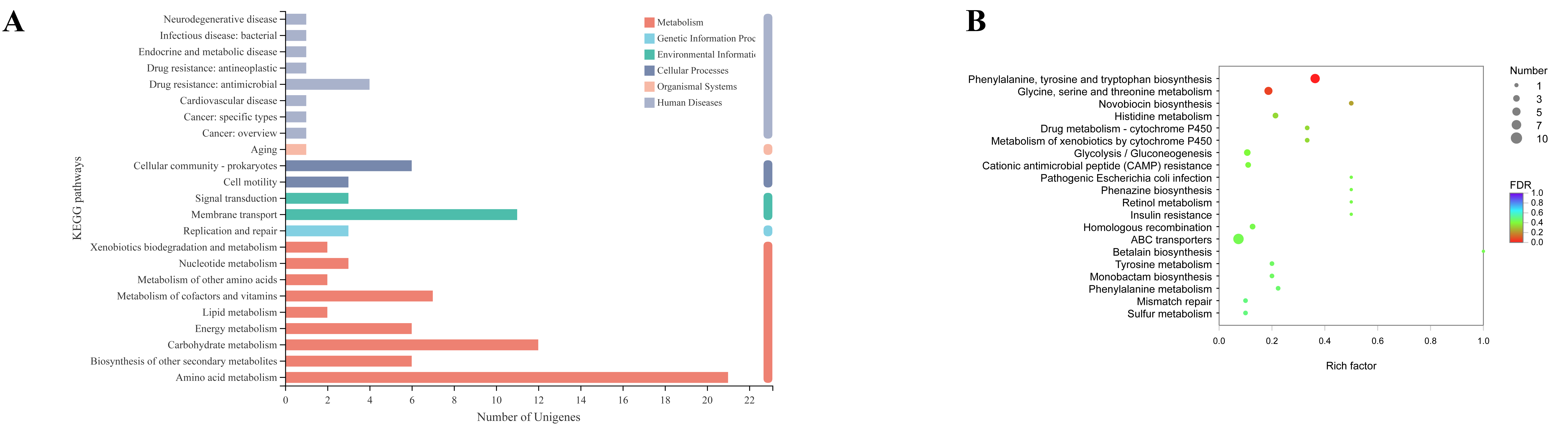

Supplement: Supplementary file 1 [file microorganisms-10-02033-s001.zip › microorganisms-1875019-supplementary/Supplemental Materials/Figure S8.tif]

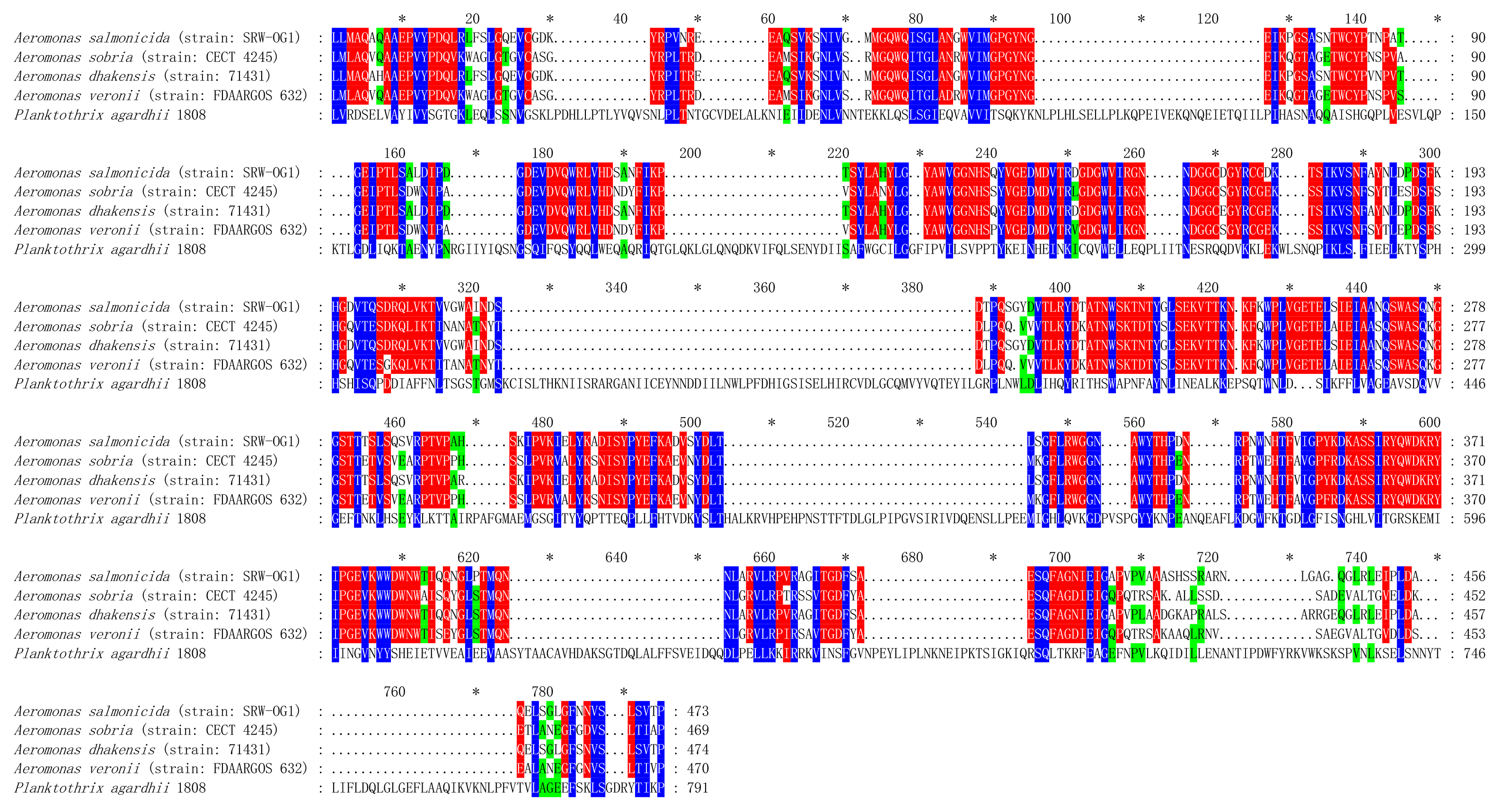

Supplement: Supplementary file 1 [file microorganisms-10-02033-s001.zip › microorganisms-1875019-supplementary/Supplemental Materials/Figure S9.tif]
